# Systems metabolic engineering of *Escherichia coli* for hyper-production of 5‑aminolevulinic acid

**DOI:** 10.1186/s13068-023-02280-9

**Published:** 2023-02-24

**Authors:** Wei Pu, Jiuzhou Chen, Yingyu Zhou, Huamin Qiu, Tuo Shi, Wenjuan Zhou, Xuan Guo, Ningyun Cai, Zijian Tan, Jiao Liu, Jinhui Feng, Yu Wang, Ping Zheng, Jibin Sun

**Affiliations:** 1 Key Laboratory of Systems Microbial Biotechnology, Chinese Academy of Sciences, Tianjin Institute of Industrial Biotechnology, Tianjin, 300308 China; 2 National Technology Innovation Center of Synthetic Biology, Tianjin, 300308 China; 3 University of Chinese Academy of Sciences, Beijing, 100049 China; 4 College of Biotechnology, Tianjin University of Science and Technology, Tianjin, 300457 China

**Keywords:** 5‑Aminolevulinic acid, Systems metabolic engineering, *Escherichia coli*, Synthetic sRNA, Antioxidant defense system

## Abstract

**Background:**

5-Aminolevulinic acid (5-ALA) is a promising biostimulant, feed nutrient, and photodynamic drug with wide applications in modern agriculture and therapy. Although microbial production of 5-ALA has been improved realized by using metabolic engineering strategies during the past few years, there is still a gap between the present production level and the requirement of industrialization.

**Results:**

In this study, pathway, protein, and cellular engineering strategies were systematically employed to construct an industrially competitive 5-ALA producing *Escherichia coli*. Pathways involved in precursor supply and product degradation were regulated by gene overexpression and synthetic sRNA-based repression to channel metabolic flux to 5-ALA biosynthesis. 5-ALA synthase was rationally engineered to release the inhibition of heme and improve the catalytic activity. 5-ALA transport and antioxidant defense systems were targeted to enhance cellular tolerance to intra- and extra-cellular 5-ALA. The final engineered strain produced 30.7 g/L of 5-ALA in bioreactors with a productivity of 1.02 g/L/h and a yield of 0.532 mol/mol glucose, represent a new record of 5-ALA bioproduction.

**Conclusions:**

An industrially competitive 5-ALA producing *E. coli* strain was constructed with the metabolic engineering strategies at multiple layers (protein, pathway, and cellular engineering), and the strategies here can be useful for developing industrial-strength strains for biomanufacturing.

**Supplementary Information:**

The online version contains supplementary material available at 10.1186/s13068-023-02280-9.

## Background

5‑Aminolevulinic acid (5-ALA) is the common precursor for biosynthesis of tetrapyrrole compounds such as vitamin B_12_, heme, and chlorophyll. Recently, 5-ALA is attracting increasing attention as a photodynamic drug and nutrient in medicine, and a biostimulant, herbicide, and insecticide in agriculture [1, 2]. Although natural microbial producers of 5 ALA such as algae and photosynthetic bacteria have been discovered for decades [3, 4], the production levels are relatively low and the highest titer of 5-ALA is 3.6 g/L [5]. Two 5-ALA biosynthetic pathways, the C4 pathway (condensation of succinyl‐coenzyme A and glycine) and C5 pathway (cascade reactions with glutamate as the precursor), have been discovered and characterized [6]. Since then, synthetic strains based on genetically tractable and well-studied platform microbes, mainly *Escherichia coli* and *Corynebacterium glutamicum*, have been engineered for microbial production of 5-ALA from renewable resources [7, 8].

Microbial production of 5-ALA has been improved by using metabolic engineering strategies during the past few years in engineered *E. coli* [9–11] and *C. glutamicum* strains [12, 13]. To improve 5-ALA production, previous studies cloned and optimized the heterologous expression of different ALA synthases (ALASs) [14–17], and strengthened the precursor supply pathway [13, 18–22], and down-regulated the heme biosynthesis [23–27]. To the best of our knowledge, there are few examples using systems metabolic engineering strategy to engineer strains for 5-ALA hyper-production, which results in the limited production level of 5-ALA (Additional file 1: Table S1). The highest 5-ALA production level for engineered *E. coli* is 15.6 g/L with the productivity 0.560 g/L/h [9]. Although the microbial cell factory based on *C. glutamicum* obtained a higher 5-ALA titer up to 25.1 g/L, the productivity (0.522 g/L/h) and yield (0.168 mol/mol) still need to be improved [12].

Systems metabolic engineering, which integrates systems and synthetic biology with metabolic engineering, has been proposed as an update for traditional metabolic engineering strategies. It covers multi-layer reprogramming of cellular metabolism including metabolic pathway reconstruction, metabolic flux optimization, tolerance enhancement, fermentation optimization, etc. [28, 29]. Its emergence has expedited the development of hyper-producing strains for various biochemicals and biofuels [30, 31], such as amino acids and their derivatives [32–35], polymers [36, 37], plant natural products [38, 39], and alcohols [40, 41].

In this study, we aim to de novo construct an industrially competitive 5-ALA producing strain using systems metabolic engineering strategies. Using *E. coli* MG1655 wild-type strain as a chassis, a heterogeneous ALAS was overexpressed to complete the C4 biosynthetic pathway [16]. 5-ALA bioproduction was then systematically optimized at multiple layers (protein, pathway, and cellular robustness) including engineering of the precursor supply and product degradation pathways, the biosynthetic enzyme for deregulation of feedback inhibition, and the cellular responsive systems for intracellular accumulation and cytotoxicity of 5-ALA, generating an engineered strain with the highest 5-ALA production level to date (30.7 g/L, 1.02 g/L/h, and 0.532 mol/mol).

## Results and discussion

### Down-regulation of competitive branch pathways via synthetic sRNA

**Fig. 1:**
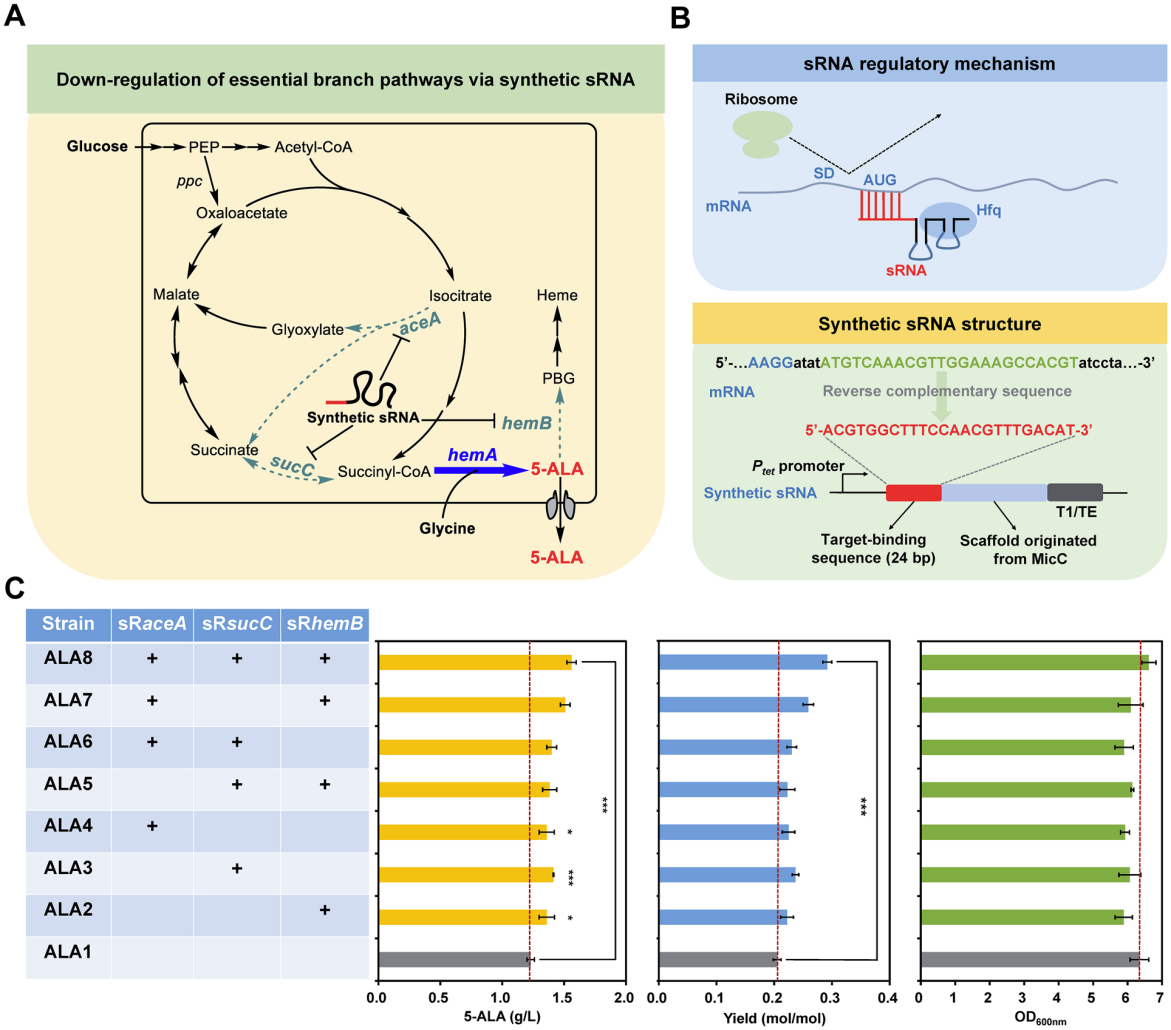
Down-regulation of competitive branch pathways via synthetic sRNA for 5-ALA production in *E. coli*. **A** Biosynthetic pathway of 5-ALA and regulation of essential branch pathways via synthetic sRNA. The dotted lines represent the target pathways for synthetic sRNA-mediated repression. The bold blue arrow represents overexpression of ALAS encoding by *hemA*. **B** The regulatory mechanism and genetic structure of synthetic sRNA. The synthetic sRNA is composed of two parts: a MicC scaffold and a target-binding sequence. The scaffold structure is responsible for recruiting the RNA chaperone Hfq protein that helps to facilitate the hybridization of sRNA and target mRNA as well as mRNA degradation. The target-binding sequence guides the synthetic sRNA to bind its target mRNA. SD means the Shine–Dalgarno sequence. T1/TE, transcriptional terminator (MITRegistry BBa_B0025). *P*_*tet*_, aTc-inducible *tet* promoter. The green mRNA sequence represents the synthetic sRNA binding site. The red sequence means a binding sequence that is complementary to the coding sequence that spans the AUG to nucleotide +21 of the target mRNA. **C** Titer, yield and cell growth of different engineered *E. coli* strains with down-regulation of essential branch pathways. (+) represents sRNA-mediated down-regulation. Data are presented as mean values +/− SD (*n* = 3 independent experiments). ***P < 0.001, *P < 0.05, Student’s two-tailed *t*-test

Succinyl-CoA, the precursor for 5-ALA biosynthesis via the C4 pathway, is located in the branching node of the tricarboxylic acid (TCA) cycle and the biosynthetic pathway of tetrapyrrole compounds (Fig. 1A). These pathways compete with each other for succinyl-CoA, resulting in a tradeoff between cell growth and 5-ALA overproduction. In order to ensure the carbon skeleton and energy supply for cell growth in the early fermentation stage, in our previous study [19], expression of ALAS was induced by adding isopropyl‐β‐d‐thiogalactopyranoside (IPTG) when the OD_600nm_ reached about 0.5 after 2.5 h of cultivation. However, due to the high flux of the TCA cycle, distribution of succinyl-CoA was usually difficult to regulate for a satisfactory 5-ALA yield. Although inactivation of succinyl-CoA synthase or succinate dehydrogenase could increase 5-ALA production, cell growth was seriously inhibited [19]. Isocitrate, a key TCA intermediate, is the branching node of the TCA cycle and glyoxylate cycle. The reaction catalyzed by isocitrate lyase (encoding by *aceA*) can bypass succinyl-CoA to produce succinate directly, which is unfavorable for 5-ALA synthesis. Moreover, the produced 5-ALA will be excessively consumed for biosynthesis of heme, an indispensable component of the respiratory chain [42]. Therefore, blocking the downstream metabolism of 5-ALA via deleting *hemB* was fatal to cells [43].

In order to reasonably allocate succinyl-CoA flux and control 5-ALA downstream metabolism without significantly affecting cell growth, essential genes involved in these branch pathways (*sucC* of TCA cycle, *aceA* of glyoxylate cycle, and *hemB* of 5-ALA degradation pathway) were down-regulated by using an anhydrotetracycline (aTc)-inducible synthetic sRNA system in a 5-ALA producing strain with *Rhodopseudomonas palustris* ALAS overexpression (Fig. 1A and B). Individual repression of *sucC*, *aceA*, and *hemB* contributed to 10.5%–15.0% increase in 5-ALA production (Fig. 1C). When three genes were combinatorially repressed, synergistic effects were produced. The 5-ALA titer and yield of strain ALA8 with simultaneous repression of *sucC*, *aceA*, and *hemB* reached 1.56 g/L and 0.292 mol/mol (Fig. 1C), which were increased by 26.8% and 38.1% compared to the control strain ALA1 without the sRNA system, respectively. These results suggest that weakening the competitive but essential branch pathways with synthetic sRNA contributes to the production of 5-ALA by properly channeling succinyl-CoA to 5-ALA biosynthesis and retarding 5-ALA downstream metabolism.

### Enhancing the supply of precursor succinyl-CoA for 5-ALA production

**Fig. 2:**
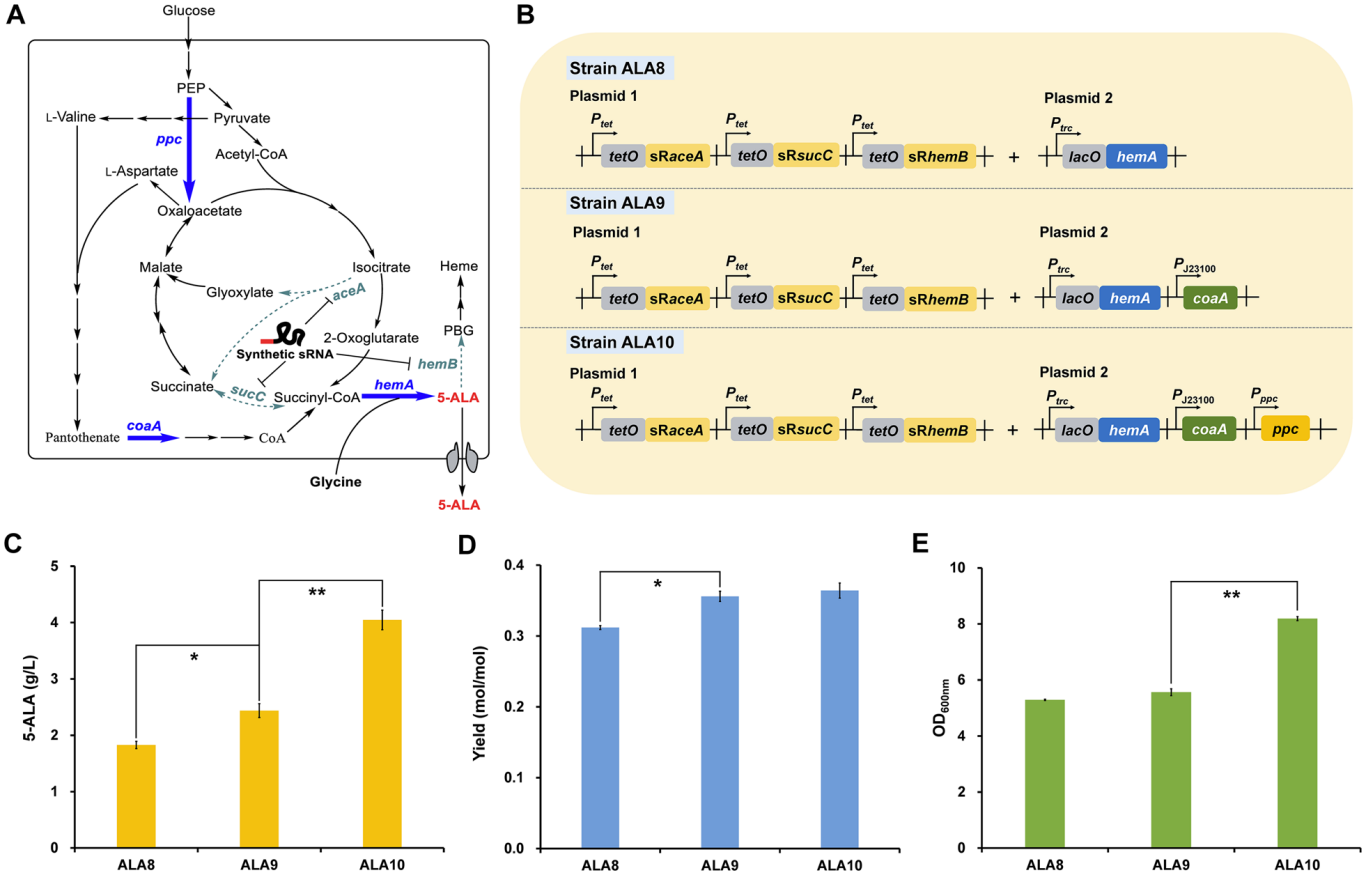
Enhancing the supply of precursor succinyl-CoA for 5-ALA production. **A** Schematic diagram of enhancing succinyl-CoA supply via overexpressing key genes (bold blue arrows). **B** Engineered strains for 5-ALA production. Genetic elements on the plasmids: *P*_*trc*_, IPTG-inducible *trc* promoter; *P*_*tet*_, aTc-inducible *tet* promoter; *P*_J23100_, J23100 constitutive promoter; *P*_*ppc*_, *ppc* promoter; *lacO*, *lac* operator; *tetO*, *tet* operator. **C** 5-ALA titer, **D** yield and **E** biomass of engineered strains. Calcium pantothenate (0.2 g/L) was supplemented. Additional 10 g/L glucose and 2 g/L glycine were supplemented after 12 h cultivation. Data are presented as mean values +/− SD (*n *= 3 independent experiments). **P < 0.01, *P < 0.05, Student’s two-tailed *t*-test

Succinyl-CoA is a thiol ester compound that is synthesized with 2-oxoglutarate and coenzyme A (CoA) as substrates. CoA is assembled in five enzymatic steps, starting from the phosphorylation of pantothenate catalyzed by pantothenate kinase (encoded by *coaA* gene) [44]. Exogenous addition of calcium pantothenate and overexpression of *coaA* could enhance the supply of intracellular CoA [45, 46]. Therefore, the effect of enhancing CoA biosynthesis on 5-ALA production was investigated (Fig. 2A). Addition of 0.2 g/L calcium pantothenate increased the 5-ALA production of strain ALA8 from 1.56 g/L to 1.83 g/L. The *coaA* gene was then overexpressed via a constitutive promoter *P*_J23100_ in strain ALA8, resulting in strain ALA9 (Fig. 2B). With calcium pantothenate addition, the titer and yield of strain ALA9 reached 2.44 g/L and 0.364 mol/mol (Fig. 2C and D), which were increased by 33.3% and 16.1% compared to the parental strain ALA8 adding calcium pantothenate, respectively. These results indicate that strengthening the supply of cofactor CoA contributes to 5-ALA biosynthesis.

Condensation of oxalacetate and acetyl-CoA is the starting point of biosynthesis of TCA intermediates including succinyl-CoA. However, the supply of oxaloacetate will be affected when succinyl-CoA is liberally used to synthesize 5-ALA. Cell growth and continuous synthesis of 5-ALA may also be limited due to the decrease of metabolic flow from Embden–Meyerhof–Parnas pathway (EMP) to TCA cycle. The four-carbon dicarboxylic acid anaplerotic pathway is an important intracellular oxalacetate complement pathway [47]. It has been demonstrated that strengthening this pathway benefits accumulation of TCA intermediate-derived chemicals, such as amino acids derived from oxaloacetate (e.g., l-lysine and l-threonine) [35, 48, 49] and organic acids (e.g., succinate and α-ketoglutarate) [50–52]. To explore the effects of reinforcing the supply of oxaloacetate on 5-ALA production, *ppc* (encoding phosphoenolpyruvate carboxylase) was overexpressed via plasmid with its own promoter* P*_*ppc*_ in strain ALA9, resulting in strain ALA10 (Fig. 2B). The 5-ALA titer was significantly increased by 66.0%, reaching 4.05 g/L (Fig. 2C). Moreover, the biomass formation was also largely increased by 47.3% compared to ALA9, even though the yield was slightly affected (Fig. 2D and E). These results suggest that enhancing the four-carbon dicarboxylic acid anaplerotic pathway can compensate for the shortage of oxaloacetate caused by 5-ALA synthesis and contribute to both cell growth and 5-ALA biosynthesis.

### Engineering of ALAS for releasing heme feedback inhibition and improving catalytic activity

Engineering of the 5-ALA biosynthetic pathway and competitive branch pathways tripled the 5-ALA production from 1.23 g/L of strain ALA1 to 4.05 g/L of strain ALA10. With the enforcement of 5-ALA biosynthesis, the intracellular heme level may also be elevated [24, 53]. ALAS is the key enzyme for 5-ALA biosynthesis but its activity is commonly inhibited by heme [54]. Therefore, releasing the feedback inhibition of ALAS by heme is expected to further improve 5-ALA production. Previous studies have shown that heme-regulatory proteins usually have specific heme-regulatory sequence, which uses Cys-Pro (CP) motif or independent l-cysteine as the axial ligand to interact with the iron in the center of heme to form heme–protein complex and regulate protein activity [55]. There are generally multiple CP motifs in the ALASs from eukaryotes (such as mice and yeast) [55]. However, no typical CP motif has been found in ALASs from prokaryotes. Therefore, l-cysteine residues are selected as the targets for engineering.

**Fig. 3:**
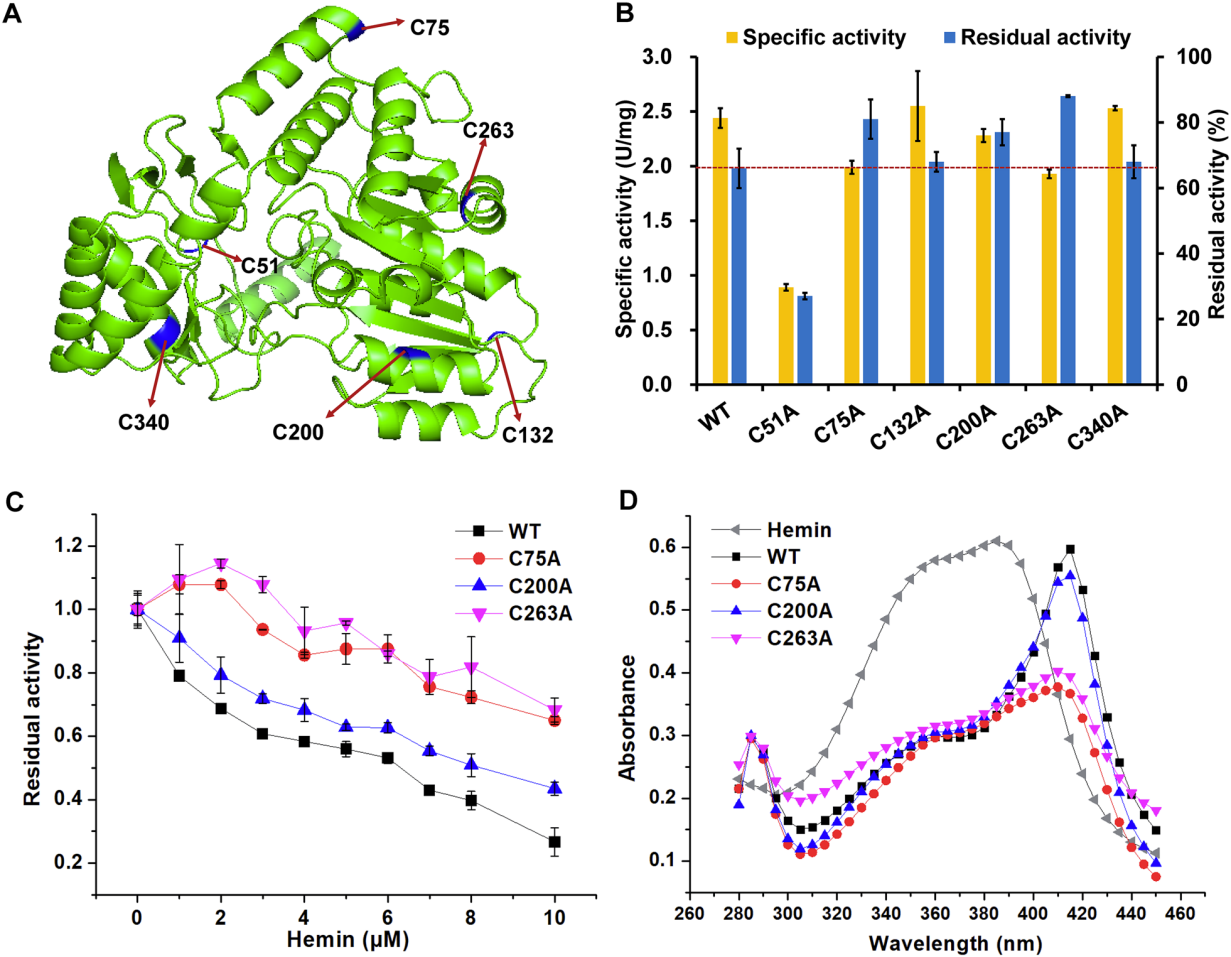
Protein engineering of ALAS for deregulation of heme inhibition. **A** The l-Cysteine residues in ALAS (PDB ID: 7X98). The C51, C75, C132, C200, C263, and C340 residues in the protein structure are indicated in blue. **B** The specific activities and residual activities of wild-type ALAS and its mutants with or without adding 2.5 µM hemin. Residual activity was determined after incubating the enzyme with 2.5 µM hemin at 37 ℃ for 1 h. Residual activity was reported as a percentage of the activity measured without hemin. **C** The residual activities of wild-type ALAS and its mutants with hemin (0 µM to 10 µM). **D** The UV–Vis spectra of the mixture of 20 µM hemin and 10 µM ALAS. The mixture was chilled on ice for 1 h and used for full-wavelength scanning. Data are presented as mean values +/− SD (*n* = 3 independent experiments)

We have characterized the crystal structure of ALAS from *R. palustris* 17001 (encoded by *hemA*) used in this study [56]. There are six l-cysteine residues (C51, C75, C132, C200, C263 and C340), which locate on the surface of ALAS and do not form intramolecular or intermolecular disulfide bonds (Fig. 3A). Therefore, these six l-cysteine residues have the possibility of specific binding with heme. To verify the hypothesis, the l-cysteine residues were individually mutated to l-alanine. In the absence of hemin (heme chloride, the purified form of natural heme that is commercially available), the specific activities of C132A and C340A mutants were almost the same as that of the wild-type ALAS, while those of other mutants were decreased by different degrees (Fig. 3B). With addition of 2.5 µM hemin, the activity of the wild-type ALAS was decreased by 34.0%. The residual activities of the C75A, C200A, and C263A mutants were 81.4%, 77.0%, and 88.4%, which were 1.23-fold, 1.17-fold and 1.33-fold higher than that of the wild-type ALAS, respectively (Fig. 3B). The other mutants (C51A, C132A, and C340A) showed severer or similar inhibition by hemin compared with the wild-type ALAS. The effects of hemin on catalytic activity were characterized in detail for the three best mutants (C75A, C200A, and C263A). For all the tested hemin concentrations, the three mutants showed lower sensitivity than the wild-type ALAS. In the presence of 10 µM hemin, the residue activities of the C75A, C200A, and C263A mutants were 2.71-, 1.79-, and 2.83-fold higher than that of the wild-type ALAS, respectively (Fig. 3C). Moreover, the UV–Vis absorption spectroscopy was used to determine whether the binding ability between hemin and ALAS was altered by the mutations. The free hemin exhibited a spectrum with a broad absorption peak from 350 to 390 nm, while the peak of hemin–ALAS complex was shifted to around 415 nm (Fig. 3D), which were consistent with a previous study [55]. The peaks of hemin–ALAS^C75A^ and hemin–ALAS^C263A^ significantly decreased, while that of hemin–ALAS^C200A^ slightly decreased, suggesting weakened binding between hemin and ALAS^C75A^ or hemin–ALAS^C263A^. The observation agrees with the alleviative inhibition of hemin caused by the C75A and C263A mutations (Fig. 3C and D).

**Fig. 4:**
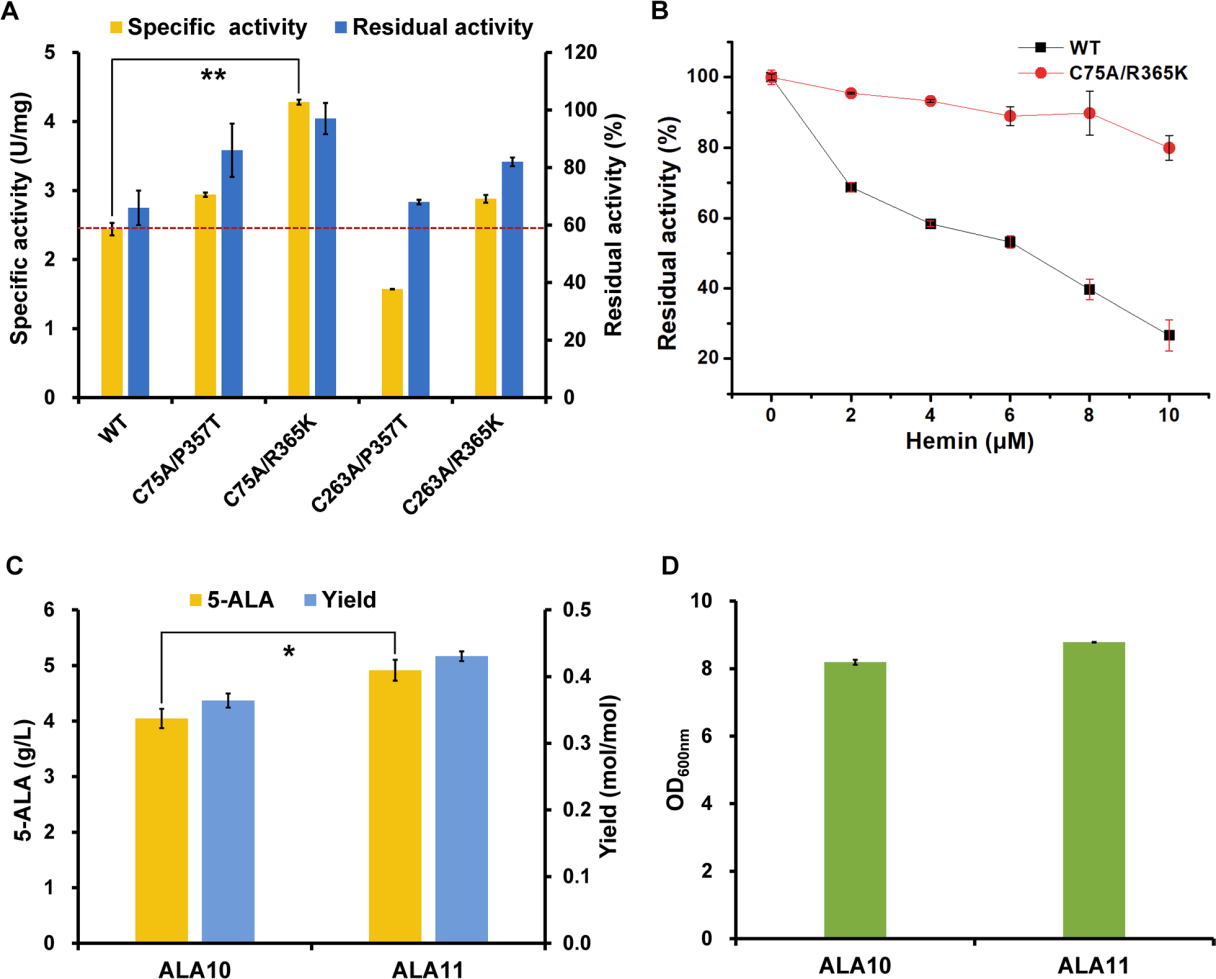
Protein engineering of ALAS for releasing heme inhibition and improving catalytic activity. **A** Specific activities and residual activities with hemin of ALAS and its double-site mutants. Residual activity was determined after incubating the enzyme with 2.5 µM hemin at 37 ℃ for 1 h. Residual activity was reported as a percentage of the activity measured without hemin. **B** Inhibition curve of wild-type ALAS and mutant C75A/R365K with different concentration of hemin. **C** 5-ALA titer, yield and **D** biomass of strains overexpressing the wild-type ALAS (strain ALA10) and ALAS^C75A/R365K^ mutant (strain ALA11): replacement the wild-type *hemA* gene of ALA10 to mutant *hemA*^C75A/R365K^ gene. Data are presented as mean values +/− SD (*n* = 3 independent experiments). **P < 0.01, *P < 0.05, Student’s two-tailed *t*-test

Although the C75A and C263A mutants showed significantly alleviative feedback inhibition of hemin, their specific activities decreased compared to the wild-type ALAS. Further engineering of ALAS to resume its catalytic activity is required. A previous study on murine ALAS2 showed that mutating some amino acid residues of the active site loop, such as R433K and A425T, improved the catalytic activity [57]. In order to resume the catalytic activity of C75A and C263A mutants, R365K and P357T mutations corresponding to R433K and A425T in murine ALAS2, respectively, were introduced into ALAS used in this study, resulting in four double-site mutants C75A/R365K, C75A/P357T, C263A/R365K, and C263A/P357T. The specific activities of these mutants, except for the C263A/P357T mutant, were significantly enhanced compared with the wild-type ALAS. Especially, the specific activity of the C75A/R365K mutant was 1.75-fold higher than that of the wild-type ALAS (Fig. 4A). At the same time, the C75A/R365K mutant showed good resistance to hemin and retained 96.6% residual activity with 2.5 µM hemin and 79.9% residual activity with 10 µM hemin. Its residual activity in the presence of 10 µM hemin was 2.96-fold higher than that of the wild-type ALAS (Fig. 4B).

Finally, in order to test whether the C75A/R365K mutant is beneficial for 5-ALA accumulation in vivo, it was introduced into strain ALA10, resulting in strain ALA11. The 5-ALA titer and yield of strain ALA11 were 4.91 g/L and 0.430 mol/mol (Fig. 4C), which were increased by 21.2% and 18.1% compared to the parental strain ALA10, respectively. This suggests that releasing the feedback inhibition of ALAS by heme and improving its catalytic activity are beneficial for 5-ALA production.

ALAS is the key enzyme for 5-ALA biosynthesis, and its enzymatic property directly determines the efficiency of 5-ALA biosynthesis. Previous studies have cloned and identified ALASs from a variety of species and used them for the construction of 5-ALA producing strains. However, most of the natural ALAS suffers from low catalytic efficiency and/or serious feedback inhibition by heme [16, 58–60], which would limit their practical application in bioproduction of 5-ALA (Zhang et al. [16]). Tan et al. obtained two *R. palustris* ALAS variants (H29R and H15K) with moderately enhanced thermostability and released feedback inhibition via computer-aided rational enzyme engineering. However, overexpression of the mutants only slightly improved 5-ALA production [54]. In this study, combining l-cysteine substitution and activity recovery mutations successfully produced an ALAS mutant with higher catalytic activity and significantly released feedback inhibition. Besides, overexpression of this mutant supported high-level 5-ALA production.

### Cellular engineering of *E. coli* for enhanced resistance to 5-ALA

**Fig. 5:**
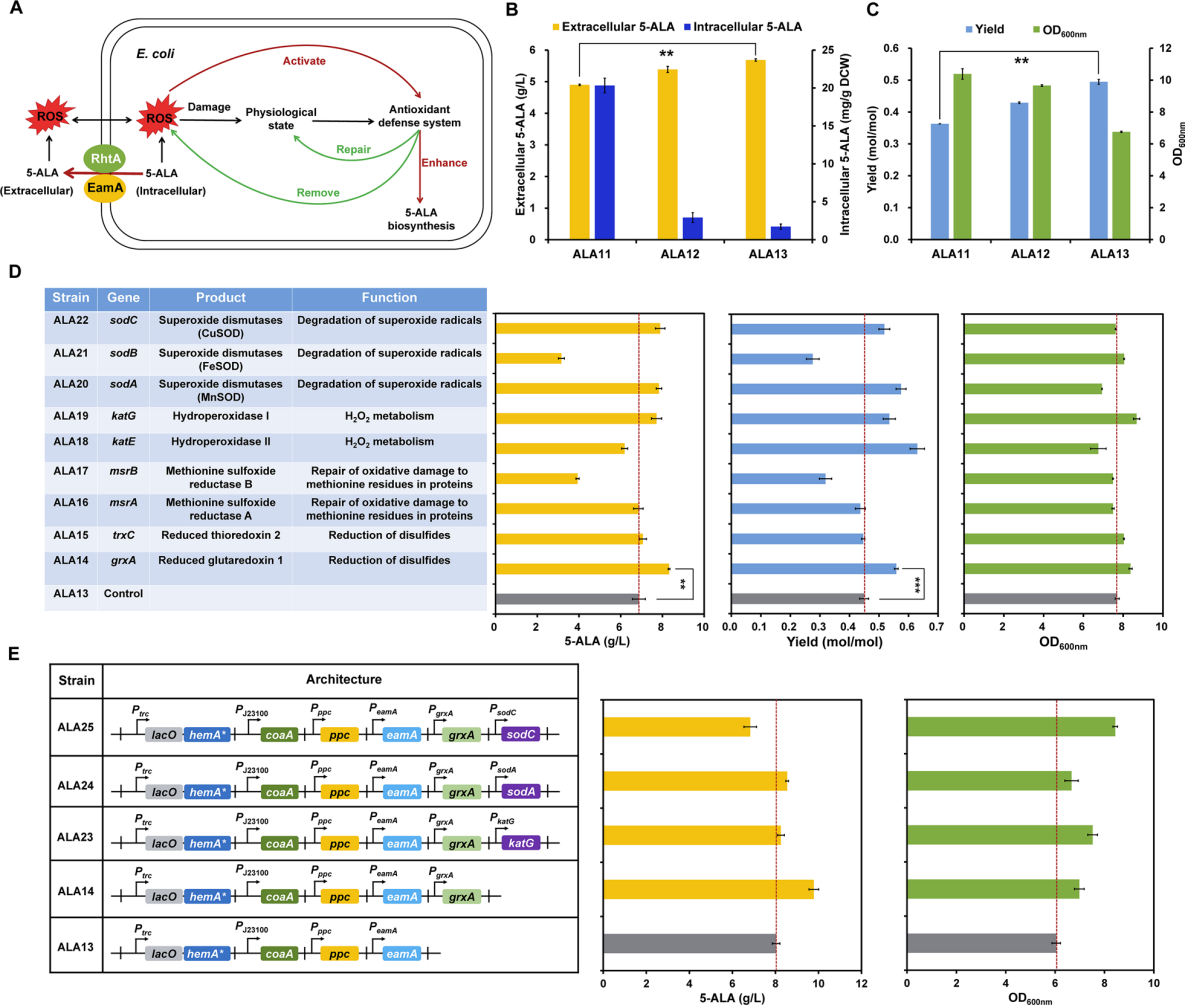
Cellular engineering of *E. coli* for enhanced resistance to 5-ALA. **A** Generation and remove of ROS caused by intracellular and extracellular 5-ALA accumulation. **B** Intracellular and extracellular 5-ALA concentrations of strains overexpressing *rhtA* or *eamA*. **C** Yield and cell growth of strains overexpressing *rhtA* or *eamA*. Strains ALA12 and ALA13 represent strain ALA11 derivatives overexpressing *rhtA* and *eamA* gene, respectively. **D** Effects of reinforcing the antioxidant defense or oxidative damage repair systems on 5-ALA production. Control represents strain ALA13. The rest strains represent strain ALA13 derivatives overexpressing the gene of antioxidant defense and oxidative damage repair system, respectively. **E** Effects of simultaneously reinforcing the antioxidant defense and oxidative damage repair systems on 5-ALA production. All engineered strains contain the plasmid overexpressing the synthetic sRNAs targeting *sucC*, *aceA*, and *hemB*. Data are presented as mean values +/− SD (*n* = 3 independent experiments). **P < 0.01, Student’s two-tailed *t*-test

5-ALA causes severe cell damage and morphology change of *E. coli* by generating reactive oxygen species (ROS) [11]. Excess ROS will cause oxidative injury to proteins, DNAs, and lipids, consequently inhibiting cellular metabolism. In order to combat with the toxicity of 5-ALA, it is necessary to enhance the excretion of intracellular 5-ALA and accelerate the removal of ROS, which should further promote production of 5-ALA (Fig. 5A).

The rapid excretion of target metabolites is not only beneficial to the accumulation of extracellular products, but also can prevent the intracellular degradation of products and reduce the toxicity to cells. Until now, no specific 5-ALA exporter has been discovered [61], whereas RhtA, a transporter with low substrate specificity was capable of excreting 5-ALA [17]. RhtA belongs to the superfamily of drug/metabolite transporters. Overexpression of RhtA increased production of l-threonine, l-homoserine, l-lysine and l-proline in the respective producing strains, which indicates its broad substrate spectrum [62]. We noticed that another member of this superfamily, EamA, was capable of excreting metabolites of the l-cysteine and l-threonine biosynthetic pathways [62, 63], and had overlapped substrate spectrum with RhtA. Therefore, we speculated that EamA may also serve as a 5-ALA exporter. The *rhtA* and *eamA* genes of *E. coli* were individually overexpressed with their own promoters in the producing strain ALA11, resulting in strains ALA12 and ALA13, respectively. The accumulation of intracellular and extracellular 5-ALA in the engineered strains were detected. As shown in Fig. 5B, the titer of extracellular 5-ALA of ALA12 and ALA13 was 5.39 g/L and 5.69 g/L, improved by 10.0% and 16.1% compared with the parental strain ALA11, respectively. Meanwhile, the intracellular 5-ALA level decreased by 85.6% and 91.5%, respectively (Fig. 5B). The yield of strains ALA12 and ALA13 was also increased by 19.4% and 36.1%, reaching 0.429 mol/mol and 0.495 mol/mol, respectively (Fig. 5C). The enhanced 5-ALA production also caused lower biomass formation, suggesting that rapid excretion of 5-ALA drove the metabolic flux from cell growth to product biosynthesis (Fig. 5C).

Strengthening the transport system of target compounds has proved to be an effective strategy of systems metabolic engineering to improve the production of many bio-based chemicals. Therefore, identification of transporters has always been considered important for developing microbial cell factories [64, 65]. In this study, overexpression of EamA led to lower intracellular 5-ALA concentration and higher extracellular 5-ALA production compared with overexpression of RhtA, manifesting that EamA is a novel exporter of 5-ALA, and its transport capacity is superior to that of RhtA.

Even though the intracellular 5-ALA is transported out of cells, extracellular accumulation of high-level 5-ALA may also generate excessive ROS and exert oxidative injury to cells (Fig. 5A). The native antioxidant defense and oxidative damage repair systems were then activated to improve cellular tolerance to 5-ALA and ultimately enhance 5-ALA production (Fig. 5A). Genes that have been reported to be involved in oxidative damage repair (*grxA*, *trxC*, *msrA*, and *msrB*) and ROS clearance (*katE*, *katG*, *sodA*, *sodB*, and *sodC*) were systematically investigated for their influence on 5-ALA production (Fig. 5D). Regarding the genes related to oxidative damage repair (*grxA*, *trxC*, *msrA*, and *msrB*), only overexpression of the *grxA* gene increased 5-ALA production by 21.1% and the titer reached 8.32 g/L 5-ALA compared with the control strain ALA13 (Fig. 5D). As for the genes related to ROS clearance, by overexpression of *katG*, *sodA*, and *sodC*, the titer of 5-ALA reached 7.72 g/L, 7.83 g/L and 7.89 g/L, increased by 12.4%, 14.0%, and 14.9% compared with the control strain ALA13, respectively (Fig. 5D). To test the combinational effect of oxidative damage repair system and ROS clearance system on 5-ALA production, three effective genes, *katG*, *sodA*, and *sodC*, were individually expressed in the strain ALA14 overexpressing *grxA*, generating strains ALA23, ALA24, and ALA25, respectively. Unfortunately, these combinations did not further improve 5-ALA production (Fig. 5E). These results show that enhancement of cellular tolerance to 5-ALA by activating the ROS clearance system or oxidative damage repair system benefits 5-ALA production.

Our previous work has demonstrated that overexpression of ROS clearance genes (*katE*, *katG*, *sodA*, *sodB*, and *sodC*) led to different levels of improvement in 5-ALA tolerance and production [11]. Different from our previous observation, overexpression of *katE* and *sodB* did not benefit 5-ALA production in this study. Such difference may be due to the different genetic backgrounds and 5-ALA production levels of the tested strains. In our previous study, only an ALAS was expressed in *E. coli* and 5-ALA titer was relatively low [11], whereas multiple genes have been engineered for enhanced 5-ALA production in the present study. Besides, effects of genes involved in oxidative damage repair on 5-ALA synthesis have not been studied. Herein, we found that reinforcing the oxidative damage repair system by overexpressing *grxA* (encoding the reduced glutaredoxin responsible for catalyzing the reduction of disulfides) was even more effective for enhancing 5-ALA production, indicating that the oxidative damage caused by 5-ALA overproduction was the key limitation for biosynthesis of 5-ALA. These results also suggest that the ROS and oxidative damages produced in different strains are different, and appropriate clearance and repair systems are required to maintain cell activity and 5-ALA production. Considering that many bio-based chemicals including lipids, *n*‐butanol, and ethanol will cause oxidative stress to the hosts [66–68], this study provides useful strategies for optimizing cellular tolerance and production of target chemicals.

### Fed-batch fermentation for 5-ALA hyper-production using the systematically engineered *E. coli*

**Fig. 6:**
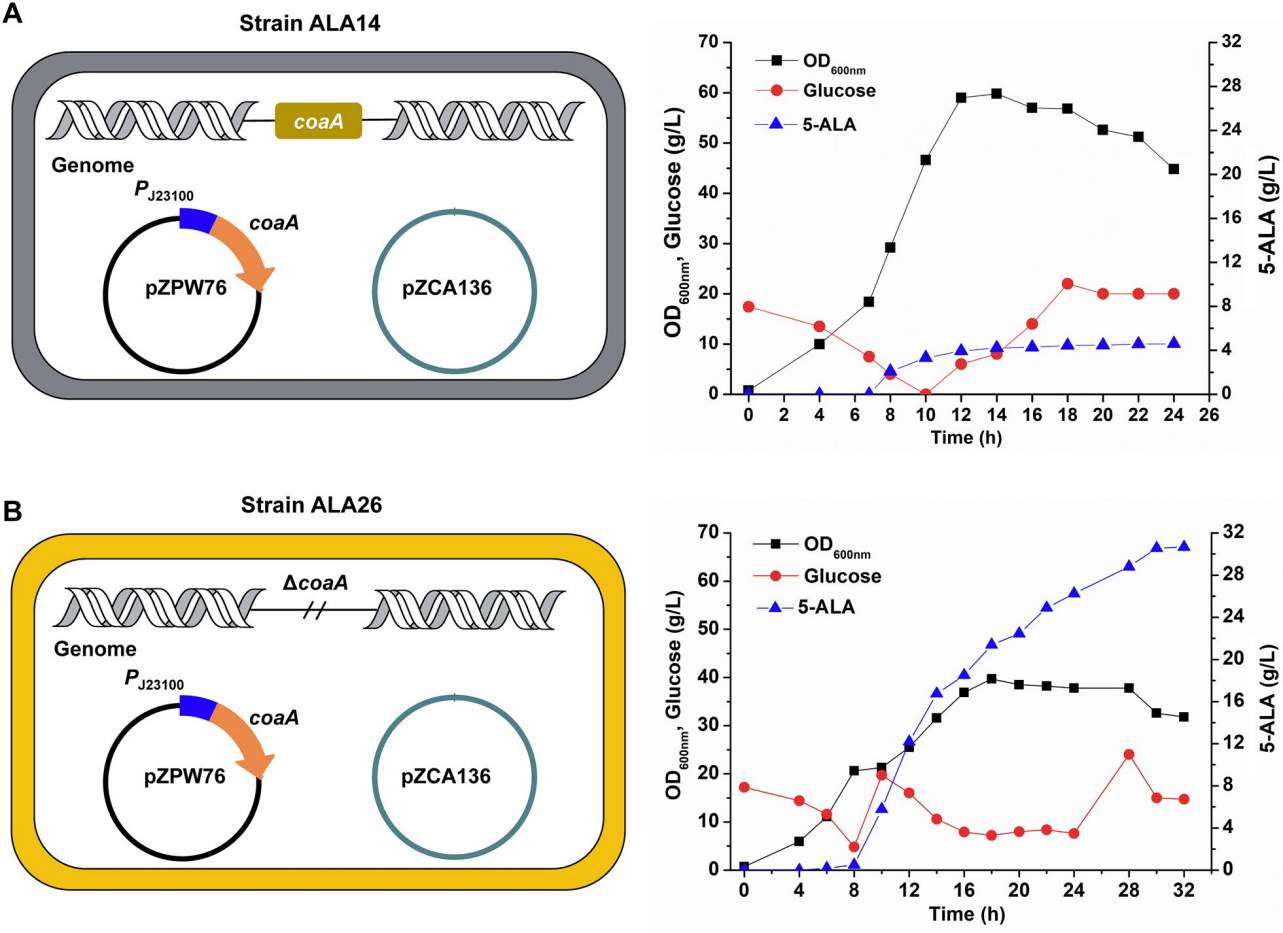
Fed-batch fermentation of strain ALA14 **(A)** and ALA26 **(B)** in 5-L bioreactors. Plasmid pZPW76 represents pTrc99A overexpressing *hemA*^C75A/R365K^, *coaA*, *ppc*, *eamA* and *grxA*. Plasmid pZCA136 represents pZCA9P overexpressing synthetic sRNAs targeting *hemB*, *sucC* and *aceA*. The chromosomal *coaA* copy was deleted in strain ALA26. Cultivation was performed in 5-L bioreactors using fermentation medium. IPTG (0.1 mM), aTc (100 ng/mL) and glycine (4 g/L) were added when OD_600nm_ reached approximately 20 to induce gene expression and 5-ALA biosynthesis. Glucose and glycine were continuously fed into the bioreactor during the fermentation. The pH was controlled at 6.5 initially and switched to 6.0 at 14 h. The dissolved oxygen and cultivation temperature were maintained at 30% and 37 ℃ initially and switched to 10% and 30 ℃ at 18 h, respectively.

Fed-batch fermentation was then conducted in 5-L bioreactors using the systematically engineered 5-ALA producing strain ALA14 that showed the best performance in 24-well plate fermentation for achieving a higher product titer. IPTG and aTc were added to induce ALAS expression and the synthetic sRNA system to initiate 5-ALA biosynthesis and weaken the competitive pathways when the OD_600nm_ reached ~20 at ~7 h. Accumulation of 5-ALA was gradually increased after induction, whereas the production ceased after 12 h (Fig. 6A). Through verification of plasmid stability, it was found that the plasmid (pZPW76) overexpressing *hemA*^C75A/R365K^, *coaA*, *ppc*, *eamA*, and *grxA* began to be lost at 8 h and was completely lost at 12 h (Additional file 1: Fig. S1), indicating the high metabolic burden and instability of the plasmid. While the other plasmid (pZCA136) overexpressing sRNA targeting *hemB*, *sucC* and *aceA* could stably exist in the cell during the fermentation.

In order to avoid the loss of plasmid, a house-keeping gene-based plasmid maintenance strategy was applied. *coaA* was an essential gene in the synthesis of CoA and the strain with deficiency of *coaA* could not grow in enriched medium (such as LB medium) [43]. Since the *coaA* gene has already been overexpressed in the plasmid, knock-out of the chromosomal copy of *coaA* is expected to produce a strain whose growth relies on the plasmid-borne *coaA*. If the plasmid overexpressing *coaA* gene is lost during fermentation, the cell cannot grow and will be weeded out from the culture (Fig. 6B). Therefore, the *coaA* of strain ALA14 was knocked out, resulting in strain ALA26. Fed-batch fermentation was then conducted in 5-L bioreactors using strain ALA26. After induction, 5-ALA biosynthesis was initiated, and the extracellular 5-ALA accumulation continued to increase until the end of fermentation at 30 h. Although cell growth was reduced, the final titer of 5-ALA reached 30.7 g/L with a productivity of 1.02 g/L/h and a yield of 0.532 mol/mol (Fig. 6B). Verification of the plasmid stability showed that both plasmids were stably maintained during the fermentation (Additional file 1: Fig. S2). These results suggest that the house-keeping gene-based plasmid maintenance strategy is beneficial for strain stability and 5-ALA production, and maybe a universal strategy for maintaining the stability of other plasmid-containing hyper-producing strains of bio-based chemicals. The systematically engineered strain ALA26 is an efficient 5-ALA producer with the highest titer, yield and productivity ever reported, and holds great potential for industrial application.

## Conclusions

In conclusion, an industrially competitive 5-ALA producing *E. coli* strain was constructed through pathway engineering to strengthen the precursor supply and reduce product degradation, protein engineering to release feedback inhibition and improve catalytic activity of the key biosynthetic enzyme, transport engineering to enhance 5-ALA excretion, and tolerance engineering to combat with 5-ALA cytotoxicity. The best strain achieved the highest 5-ALA production level (30.7 g/L) in a 5-L bioreactor with a productivity of 1.02 g/L/h and a yield of 0.532 mol/mol. The strategies presented here can be applied to other microorganisms for the production of valuable chemicals.

## Material and methods

### Bacterial strains and growth conditions

*Escherichia coli* strains used in this study are listed in Additional file 1: Table S2. *E. coli* DH5α was used for general cloning. *E. coli* MG1655 was employed as a host for 5-ALA production. *E. coli* BL21 (DE3) was used for expression of ALAS and its mutants. For routine cultivations including plasmid and strain construction, *E. coli* strains were cultivated at 37 °C and with shaking at 220 rpm in Luria–Bertani (LB) broth. Agar was added at 1.5% w/v for LB plates. Ampicillin (Amp, 100 μg/mL), chloramphenicol (Cm, 20 µg/mL) or anhydrotetracycline (aTc, 100 ng/mL) were added as required.

### Plasmid construction

Plasmids and primers used in this study are listed in Additional file 1: Tables S2 and S3. Primer synthesis and Sanger sequencing were performed by GENEWIZ (Suzhou, China). All enzymes were obtained from Vazyme (Nanjing, China) or TransGen (Beijing, China). Unless otherwise stated, plasmids were constructed via recombination by using the ClonExpress MultiS One Step Cloning Kit (Vazyme, Nanjing, China).

For the construction of plasmids pZPA18 and pZPA19, the *coaA* gene and its upstream 500 bp and downstream 500 bp fragments were amplified from the genomic DNA of *E. coli* MG1655 using *TransStart® FastPfu* DNA Polymerase (TransGen, Beijing, China) with the primer pair *coaA-*500-F/*coaA*-500-R. The PCR product was ligated with the plasmid pEASY-Blunt (TransGen, Beijing, China), resulting in plasmid pEASY-Blunt-500-*coaA*-500 (pZPA17). The backbone of plasmid pZPA17 was amplified by PCR with the primer pair *coaA*-Rev-F/*coaA*-Rev-R to remove the *coaA* gene. The backbone of plasmid pZPA17 was digested with *Dpn* I, phosphorylated with T4 polynucleotide kinase, and self-ligated using T4 DNA ligase, resulting in the plasmid pEASY-Blunt-500–500 (pZPA18). The fragment containing the chloramphenicol-resistant gene (*cm*) and the sucrose counter-selectable gene (*sacB*) was amplified from the plasmid pDS132 [69] using *TransStart® FastPfu* DNA Polymerase (TransGen, Beijing, China) with the primer pair *cm-*F/*sacB*-R. The backbone of plasmid pZPA17 was digested with *Dpn* I, phosphorylated with T4 polynucleotide kinase, and ligated with the *cm-sacB* fragment using T4 DNA ligase, resulting in the plasmid pEASY-Blunt-500-*cm-sacB*-500 (pZPA19).

For the construction of plasmid pKD46-*P*_*coaA*_*-coaA* (pZPA43), the *coaA* promoter and *coaA* gene were amplified from the genomic DNA of *E. coli* MG1655 using *TransStart® FastPfu* DNA Polymerase (TransGen, Beijing, China) with the primer pair pKD46*-coaA-*F*/*pKD46*-coaA-*R, and were then phosphorylated with T4 polynucleotide kinase*.* The backbone of plasmid pKD46 [70] was amplified by PCR with the primer pair pKD46-Rev-F/pKD46-Rev-R. Then these two DNA fragments were purified and ligated using the CloneExpress® MultiS One Step Cloning Kit (Vazyme Biotech, Nanjing, China) to generate the plasmid pKD46-*P*_*coaA*_*-coaA* (pZPA43).

For the construction of plasmid pTrc99A-*hemA*-*P*_J23100_*-coaA* (pZPW70), the *P*_J23100_ promoter (5′-TTGACGGCTAGCTCAGTCCTAGGTACAGTGCTAGC-3′) and *coaA* gene were amplified from the genomic DNA of *E. coli* MG1655 using *TransStart® FastPfu* DNA Polymerase (TransGen, Beijing, China) with the primer pair *coaA-*F/*coaA*-R. The backbone of plasmid pTrc99A-*hemA* harboring the ALAS gene (*hemA*) from *R. palustris* 17001 [16] was amplified by PCR with the primer pair pTrc99A-Rev-2F/pTrc99A-Rev-2R. Then these two DNA fragments were purified and ligated using the CloneExpress® MultiS One Step Cloning Kit (Vazyme Biotech, Nanjing, China) to generate the plasmid pTrc99A-*hemA*-*P*_J23100_*-coaA* (pZPW70).

For the construction of plasmid pTrc99A-*hemA*-*P*_J23100_*-coaA-P*_*ppc*_*-ppc* (pZPW71), the *ppc* promoter and *ppc* gene were amplified from the genomic DNA of *E. coli* MG1655 using *TransStart® FastPfu* DNA Polymerase (TransGen, Beijing, China) with the primer pair *ppc*-F/*ppc*-R. The *ppc* gene fragment and the vector pTrc99A-*hemA*-*P*_J23100_*-coaA* were digested with restriction enzyme *Hin*d III, and the fragment of pTrc99A-*hemA*-*P*_J23100_*-coaA* was dephosphorylated with dephosphorylase. After purification of the above two DNA fragments, T4 DNA ligase was used to ligate the two gene fragments, resulting in plasmid pTrc99A-*hemA*-*P*_J23100_*-coaA-P*_*ppc*_*-ppc* (pZPW71).

For the construction of plasmid pTrc99A-*hemA*^C75A/R365K^-*P*_J23100_*-coaA-P*_*ppc*_*-ppc* (pZPW72), two rounds of site-directed mutagenesis of *hemA* were realized by using a modified QuikChange protocol [71] and with the primer pair C75A-F/C75A-R and R365K-F/R365K-R listed in Additional file 1: Table S3, respectively.

For the construction of plasmid pTrc99A-*hemA*^C75A/R365K^-*P*_J23100_*-coaA-P*_*ppc*_*-ppc-P*_*rhtA*_*-rhtA* (pZPW73) and pTrc99A-*hemA*^C75A/R365K^-*P*_J23100_*-coaA-P*_*ppc*_*-ppc-P*_*eamA*_*-eamA* (pZPW74), the* P*_*rhtA*_ promoter and *rhtA* gene (or the *P*_*eamA*_ promoter and *eamA* gene) were amplified from the genomic DNA of *E. coli* MG1655 by PCR using *TransStart® FastPfu* DNA Polymerase (TransGen, Beijing, China) with the primer pair *rhtA-*F/*rhtA*-R (or *eamA-*F/*eamA*-R). The first part of plasmid backbone (*hemA*^C75A/R365K^ and *ppc* gene) of pZPW72 was amplified by PCR with the primer pair *hemA*-F/*ppc*-R. The second part of plasmid backbone (*bla* gene, *coaA* gene*, lacI* gene and pBR322 origin) of pZPW72 was amplified by PCR with the primer pair *amp*-F/*lacI*-R. Then these three DNA fragments were purified and ligated using the CloneExpress® MultiS One Step Cloning Kit (Vazyme Biotech, Nanjing, China) to generate the plasmid pTrc99A-*hemA*^C75A/R365K^-*P*_J23100_*-coaA-P*_*ppc*_*-ppc-P*_*rhtA*_*-rhtA* (pZPW73) and pTrc99A-*hemA*^C75A/R365K^-*P*_J23100_*-coaA-P*_*ppc*_*-ppc-P*_*eamA*_*-eamA* (pZPW74).

For the construction of overexpression plasmid of *grxA* (pZPW76), the *P*_*grxA*_ promoter and *grxA* gene were amplified from the genomic DNA of *E. coli* MG1655 by PCR with the primer pair *grxA-*F/*grxA*-R. The *P*_*eamA*_ promoter and *eamA* gene were amplified from the genomic DNA of *E. coli* MG1655 by PCR with the primer pair *eamA-*F/*eamA*-2R. The first part of pZPW72 backbone (*hemA*^C75A/R365K^ and *ppc* gene) was amplified by PCR with the primer pair *hemA*-F/*ppc*-R, and the second part of pZPW72 backbone (*bla* gene, *coaA* gene, *lacI* gene and pBR322 origin) was amplified by PCR with the primer pair *amp*-F/*lacI*-R. Then these four DNA fragments were purified and ligated using the CloneExpress® MultiS One Step Cloning Kit (Vazyme Biotech, Nanjing, China) to generate the plasmid pTrc99A-*hemA*^C75A/R365K^-*P*_J23100_*-coaA-P*_*ppc*_*-ppc-P*_*eamA*_*-eamA-P*_*grxA*_*-grxA* (pZPW76). Construction process of overexpression plasmid of other antioxidant genes was similar to that of pZPW76, just replacing the *grxA* gene with the target antioxidant gene and its own promoter.

For the construction of plasmid co-overexpressing the antioxidant genes (pZPW85), the *P*_*eamA*_ promoter and *eamA* gene were amplified from the genomic DNA of *E. coli* MG1655 by PCR with the primer pair *eamA-*F/*eamA*-2R. The *P*_*katG*_ promoter and *katG* gene were amplified from the genomic DNA of *E. coli* MG1655 by PCR with the primer pair *katG-*F/*katG*-2R. The *P*_*grxA*_ promoter and *grxA* gene were amplified from the genomic DNA of *E. coli* MG1655 by PCR with the primer pair *grxA-2*F/*grxA*-2R. The first part of pZPW72 backbone (*hemA*^C75A/R365K^ and *ppc* gene) was amplified by PCR with the primer pair *hemA*-F/*ppc*-R, and the second part of pZPW72 backbone (*bla* gene, *coaA* gene, *lacI* gene and pBR322 origin) was amplified by PCR with the primer pair *amp*-F/*lacI*-R. Then these five DNA fragments were purified and ligated using the CloneExpress® MultiS One Step Cloning Kit (Vazyme Biotech, Nanjing, China) to generate the plasmid pTrc99A-*hemA*^C75A/R365K^-*P*_J23100_*-coaA-P*_*ppc*_*-ppc-P*_*eamA*_*-eamA-P*_*grxA*_*-grxA*-*P*_*katG*_*-katG* (pZPW85). The construction process of plasmids pZPW86 and pZPW87 was similar to that of pZPW85, just replacing the *katG* gene with *sodA* gene (or *sodC* gene) and its own promoter.

### Construction of the *coaA*-deleted strain

Deletion of the *coaA* gene in *E. coli* MG1655 was performed by the method established by Datsenko and Wanner [70] and Philippe et al. [69]. The two DNA fragments for recombination were amplified from the plasmid pZPA18 or pZPA19 using *TransStart*® *FastPfu* DNA Polymerase (TransGen, Beijing, China) with the primer pair *coaA*-500-F/*coaA*-500-R. After two rounds of screening, the chromosomal copy of *coaA* was deleted in the strain containing plasmid pZPW43 (expressing a *coaA*), generating strain ZLEcA4.

Then the plasmids pZCA136 (expressing the synthetic small RNAs targeting *sucC*, *aceA* and *hemB*) and pZPW76 were co-transformed into the competent cell of strain ZLEcA4. The transformant was cultivated at 37 ℃ for 12 h in order to cure the plasmid pZPW43 (containing a temperature-sensitive replicon) and keep the plasmids pZCA136 and pZPW76. Finally, the strain MG1655Δ*coaA* harboring the plasmids pZCA136 and pZPW76, named as strain ALA26, was obtained.

### Protein engineering of ALAS

Site-directed mutagenesis of the ALAS encoding gene *hemA* was realized by using a modified QuikChange protocol [71] and primers listed in Additional file 1: Table S3. The PCR products were treated with *Dpn* I (New England Biolabs, USA) to eliminate the template plasmid harboring the wild-type *hemA*, and thereafter transformed into competent cells of *E. coli* DH5a. Recombinant plasmids were extracted and verified by Sanger sequencing. Expression and purification of ALAS and its mutants was performed according to the procedure described previously [54]. Recombinant *E. coli* BL21 (DE3) strains harboring *hemA*-expressing plasmids were cultivated at 37 °C in LB broth supplemented with Amp to an OD_600nm_ of 0.6–0.8. IPTG was then added to a final concentration of 0.1 mM to induce *hemA* expression. After incubated at 20 °C for another 20 h, cells were harvested, washed, and resuspended in a buffer (pH 7.5) containing 50 mM Tris–HCl, 100 mM NaCl, 30 mM imidazole, 5% glycerol (v/v). The cells were disrupted by sonication in an ice bath. The lysed cells were centrifuged at 10,000×*g* for 30 min at 4 °C and the supernatant was used for further purification. Enzymes were purified from the crude extract using a His-Trap column (GE Healthcare, USA). Pure enzymes were desalted using AmiconUltra-4 centrifugal concentrator (10 kDa), exchanged into a buffer (pH 7.5) containing 50 mM Tris–HCl, 100 mM NaCl, 5% glycerol (v/v), and stored in small aliquots at − 80 °C. Protein concentration was determined with BCA Protein Assay Kit (Thermo Fisher Scientific, USA).

### Enzyme activity assays

ALAS activity was determined as previously described [16]. The reaction mixture contained 100 mM Tris–HCl (pH 7.5), 200 mM glycine, 0.2 mM succinyl-CoA, 0.1 mM pyridoxal phosphate (PLP) and enzyme (2 μg purified ALAS or 7.5 μg crude ALAS per milliliter). After proceeding at 37 ℃ for 10 min, the reaction was terminated by the addition of 10% (v/v) trichloroacetic acid. Concentration of 5-ALA in the supernatant was determined [72]. One unit of ALAS activity was defined as the amount of enzyme required for formation of 1 μmol 5-ALA per minute under assay conditions. The effect of hemin on ALAS activity was studied by determining the residual activity at varied concentrations of hemin (0, 1, 2, 3, 4, 5, 6, 8, and 10 µM). The UV–Vis absorption spectra (300 nm–500 nm) of hemin in the absence or presence of ALASs in 50 mM Tris–HCl (pH 7.5) and 100 mM NaCl were recorded after 1 h incubation on ice. The molar ratio of hemin to ALAS was 2:1.

### Small regulatory RNA (sRNA)-mediated gene knockdown

Design of synthetic sRNA and plasmid construction were realized by the method established by Sun et al. [73]. Plasmids, primers and binding sequences of sRNAs used in this study are listed in Additional file 1: Tables S2 and S4.

For the construction of plasmid pZSA14 (expressing the synthetic sRNA targeting *sucC*), the plasmid pWSK29 [74] and pSB4C5 [75] were digested with the restriction enzyme *Nde* I and *Eco*R I to obtain the DNA fragments containing the *cm-*resistant gene and pSC101 replicon, respectively. Then these two DNA fragments were ligated with T4 DNA ligase, resulting in the plasmid pZCA9. The plasmid pZCA9 was linearized using PCR with the primer pair pZCA9-F/pZCA9-R to remove the *lac* promoter, and then the linearized DNA fragment of pZCA9 was self-ligated to generate the plasmid pZCA9P. According to the method of designing sRNA established by Yoo et al. [76], the sRNA sequence containing the inductive promoter *P*_*tet*_, the 24 nt binding sequence targeting the *sucC* gene, the MicC scaffold sequence [76] and the T1/TE terminator (MITRegistry BBa_B0025) was chemically synthesized by GENEWIZ (Suzhou, China), and cloned to pUC57 to generate pUC57-sRNA. The sRNA cassette targeting *sucC* was amplified from pUC57-sRNA using the primer pair TsR-F/TsR-R. The *tetR* gene was amplified from plasmid pACYC184 [77] with the primer pair *tetR*-F/*tetR*-R. The plasmid pZCA9P was linearized using PCR with the primer pair p-F/p-R. Then these three DNA fragments were purified and ligated via recombination to generate the plasmid pZSA14. For the construction of plasmid pZSA13 (expressing the synthetic sRNA targeting *hemB*) and pZSA41 (expressing the synthetic sRNA targeting *aceA*), the plasmid pZSA14 was linearized using PCR with the primer pair sR*hemB*-F/sR*hemB*-R and sR*aceA*-F/sR*aceA*-R, respectively, and self-ligated to generate the pZSA13 and pZSA41. For the construction of plasmid pZCA134 (expressing the synthetic sRNAs targeting *sucC* and *hemB*), the plasmid pZSA14 was linearized using PCR with the primer pair TsRBC-F/TsRBC-R. The sRNA sequence of *hemB* was amplified with the primer pair TsR-F/TsR-R from the plasmid pZSA13. Then these two PCR fragments were ligated via recombination to generate the plasmid pZCA134. For the construction of plasmid pZCA135 (expressing the synthetic sRNAs targeting *sucC* and *aceA*), the plasmid pZSA14 was linearized using PCR with the primer pair TsRAC-F/TsRAC-R. The sRNA sequence of *aceA* was amplified with the primer pair TsR-F/TsR-R from the plasmid pZSA41. Then these two PCR fragments were ligated via recombination to generate the plasmid pZCA135. For the construction of plasmid pZCA136 (expressing the synthetic sRNAs targeting *hemB*, *sucC*, and *aceA*), the plasmid pZCA134 was linearized using PCR with the primer pair TsRAC-F/TsRAC-R. The sRNA sequence of *aceA* was amplified with the primer pair TsR-F/TsR-R from the plasmid pZSA41. Then these two PCR fragments were ligated via recombination to generate the plasmid pZCA136. For the construction of plasmid pZCA137 (expressing the synthetic sRNAs targeting *hemB* and *aceA*), the plasmid pZSA14 was linearized using PCR with the primer pair TsRAB-F/TsRAB-R to remove the synthetic sRNA cassette for *sucC*, generating the plasmid pZCA137.

### Batch fermentation in 24-well plates

For 5-ALA fermentation in 24-well plates, the engineered *E. coli* strains were inoculated into 1 mL LB medium in 24-well plates containing appropriate antibiotics and incubated at 37 ℃ with shaking at 800 rpm for 10 h in INFORS Microtron (INFORS HT Multitron Pro, Switzerland). The preculture was used as a seed to inoculate 1 mL modified M9 medium in 24-well plates to an initial OD_600nm_ of 0.05. The cultures were incubated at 37 °C and with shaking at 800 rpm. After 2.5 h cultivation, IPTG (0.05 mM), aTc (100 ng/mL) and glycine (4 g/L) were added to induce ALAS expression and weaken the relevant competitive pathways. Considering that 5-ALA is unstable under high temperatures [78], the fermentation temperature was shifted to 35 °C after inducing ALAS expression. After another 9.5 h cultivation, 10–15 g/L glucose and 2–6 g/L glycine were supplemented when necessary. After total cultivation of 24 h, samples were taken to analyze the 5-ALA concentration, OD_600nm_ and residue glucose. The modified M9 medium contains 15 g/L glucose, 2 g/L yeast extract, 17.1 g/L Na_2_HPO_4_·12H_2_O, 3 g/L KH_2_PO_4_, 1 g/L NH_4_Cl, 0.5 g/L NaCl, 0.24 g/L MgSO_4_·7H_2_O, and 11 mg/L CaCl_2_. Amp (100 μg/mL) and Cm (20 µg/mL) were added at the beginning of cultivation. For cultivation of the strain overexpressing *coaA* gene, 0.2 g/L calcium pantothenate was additionally supplemented.

### Fed-batch fermentation in bioreactors

For fed-batch fermentation in 5-L bioreactors, cells at late exponential phase were used to inoculate 2 L fermentation medium with an inoculation size of 5% (v/v). The fermentation medium contains 5 g/L KH_2_PO_4_, 8 g/L NH_4_Cl, 5 g/L yeast extract, 0.5 g/L MgSO_4_, 0.2 g/L calcium pantothenate and 20 g/L glucose, which was modified based on the medium described in literature [11]. Fermentation was conducted at 37 °C, 30% dissolved oxygen (automatically adjusted with aeration and agitation rates), and pH 6.5 (automatically adjusted with H_2_SO_4_ and ammonium hydroxide). Considering that 5-ALA is unstable under high temperatures and is easily oxidized in the presence of oxygen [11, 79], the dissolved oxygen and cultivation temperature were switched to 10% and 30 ℃ at 18 h, respectively. Solutions of glucose (700 g/L) and glycine (200 g/L) were fed into the bioreactor to maintain the glucose concentration between 1 and 10 g/L, and to supplement glycine with the speed of 1–2 g/L/h. IPTG (0.02 mM) and aTc (100 ng/mL) were added to induce ALAS expression and weaken competitive pathways when the OD_600nm_ reached ~20.

### Analytical methods

Cell biomass was determined by the optical density at 600 nm (OD_600nm_) with a UV-1800 spectrophotometer (Shimadzu, Kyoto, Japan). Glucose was measured by using the SBA-40 biosensor analyzer (Institute of Biology, Shandong Province Academy of Sciences, Shandong, China) equipped with a glucose oxidase membrane. The analytical signal was given by quantifying the production of H_2_O_2_ generated by glucose oxidation. 5-ALA in the fermentation broth was determined following the method described previously [72]. Organic acids were analyzed by using HPLC according to the procedure described previously [80]. The 5-ALA yield was defined as mole of 5-ALA produced/mole of glucose consumed.

The intracellular 5-ALA was measured using the following method. Firstly, the OD_600nm_ of the culture was detected with a UV-1800 spectrophotometer (Shimadzu, Kyoto, Japan). Secondly, 100 µL 50% glycerol was added into a 1.5 mL centrifuge tube and 200 µL silicone oil was added slowly [81]. Then, appropriate amount of fermentation broth was added slowly and then centrifuged at 12,000 rpm for 2 min. Finally, the cells were removed into a clean centrifuge tube and resuspended with adding 20 mM acetic acid. Lysate (200 µL) was taken to determine the intracellular 5-ALA with the method described previously [72].

### Statistical analysis

Error bars indicate standard deviations from three parallel experiments. *P* values were generated from two-tailed *t* tests using the Microsoft Excel 2016 (Microsoft Corporation).

## Supplementary Information

**Additional file 1: Fig. S1.** Verification of plasmid stability in strain ALA14 during fed-batch fermentation in 5-L bioreactors. **Fig. S2.** Verification of plasmid stability in strain ALA26 during fed-batch fermentation in 5-L bioreactors. **Table S1.** Microbial production of 5-ALA by engineered *E. coli* and *C. glutamicum* strains via C4 biosynthetic pathway from different substrates. **Table S2.** Bacterial strains and plasmids used in this study. **Table S3.** Primers used in this study. **Table S4.** Binding sequences of sRNAs used in this study.

## Data Availability

The datasets used and analyzed during the current study are available from the corresponding author on reasonable request.

## Abbreviations

5-ALA: 5-Aminolevulinic acid
ALAS: 5-ALA synthase
IPTG: Isopropyl-β-d-thiogalactopyranoside
PLP: Pyridoxal phosphate
PPC: Phosphoenolpyruvate carboxylase
CoA: Coenzyme A
EMP: Embden–Meyerhof–Parnas
ROS: Reactive oxygen species
aTc: Anhydrotetracycline
