# Correction: Systems metabolic engineering of *Escherichia coli* for hyper-production of 5-aminolevulinic acid

**DOI:** 10.1186/s13068-023-02359-3

**Published:** 2023-06-30

**Authors:** Wei Pu, Jiuzhou Chen, Yingyu Zhou, Huamin Qiu, Tuo Shi, Wenjuan Zhou, Xuan Guo, Ningyun Cai, Zijian Tan, Jiao Liu, Jinhui Feng, Yu Wang, Ping Zheng, Jibin Sun

**Affiliations:** 1 Key Laboratory of Systems Microbial Biotechnology, Chinese Academy of Sciences, Tianjin Institute of Industrial Biotechnology, Tianjin, 300308 China; 2 National Technology Innovation Center of Synthetic Biology, Tianjin, 300308 China; 3 University of Chinese Academy of Sciences, Beijing, 100049 China; 4 College of Biotechnology, Tianjin University of Science and Technology, Tianjin, 300457 China

**Correction: Biotechnology for Biofuels and Bioproducts (2023) 16:31** 10.1186/s13068-023-02280-9

Following publication of the original article [1], it came to the attention of the authors that a sentence in the Background section was not entirely accurate. The sentence in the original version of the paper:

Although natural microbial producers of 5-ALA such as algae and photosynthetic bacteria have been discovered for decades [3, 4], they are not adopted in industrial 5-ALA production due to the relatively low titer, yield, and productivity.

Has been corrected as follows:

Although natural microbial producers of 5 ALA such as algae and photosynthetic bacteria have been discovered for decades [3, 4], the production levels are relatively low and the highest titer of 5-ALA is 3.6 g/L [5].

The authors also state that the correction does not affect the discussion or conclusions and that they sincerely apologize for the unintentional errors.

The original article has been corrected.
