# Supplementary material for: Systems metabolic engineering of Escherichia coli for hyper-production of 5‑aminolevulinic acid
Source: Biotechnol Biofuels Bioprod. 2023 Feb 24;16:31. doi: 10.1186/s13068-023-02280-9 (PMC9951541; doi:10.1186/s13068-023-02280-9)
Supplement: Supplementary file 1 — Additional file 1: Fig. S1. Verification of plasmid stability in strain ALA14 during fed-batch fermentation in 5-L bioreactors. Fig. S2. Verification of plasmid stability in strain ALA26 during fed-batch fermentation in 5-L bioreactors. Table S1. Microbial production of 5-ALA by engineered E. coli and C. glutamicum strains via C4 biosynthetic pathway from different substrates. Table S2. Bacterial strains and plasmids used in this study. Table S3. Primers used in this study. Table S4. Binding sequences of sRNAs used in this study. [file 13068_2023_2280_MOESM1_ESM.docx]

**Additional file 1**

**Systems metabolic engineering of** ***Escherichia coli* for hyper-production of 5‑aminolevulinic acid**

Wei Pu^1,2†^, Jiuzhou Chen^1,2†^, Yingyu Zhou^1,4^, Huamin Qiu^1,3^, Tuo Shi^1,2^, Wenjuan Zhou^1,2^, Xuan Guo^1,2^, Ningyun Cai^1,4^, Zijian Tan^1,2^, Jiao Liu^1,2^, Jinhui Feng^1,2^, Yu Wang^1,2,^^3*^, Ping Zheng^1,2,3*^, Jibin Sun^1,2,3^

^1^Key Laboratory of Systems Microbial Biotechnology, Chinese Academy of Sciences, Tianjin Institute of Industrial Biotechnology, Tianjin 300308, China

^2^National Technology Innovation Center of Synthetic Biology, Tianjin 300308, China.

^3^University of Chinese Academy of Sciences, Beijing 100049, China

^4^College of Biotechnology, Tianjin University of Science and Technology, Tianjin 300457, China.

*Corresponding authors.

Dr. Yu Wang

wang_y@tib.cas.cn

Dr. Ping Zheng

zheng_p@tib.cas.cn

^†^Wei Pu and Jiuzhou Chen contributed equally to this work.

**
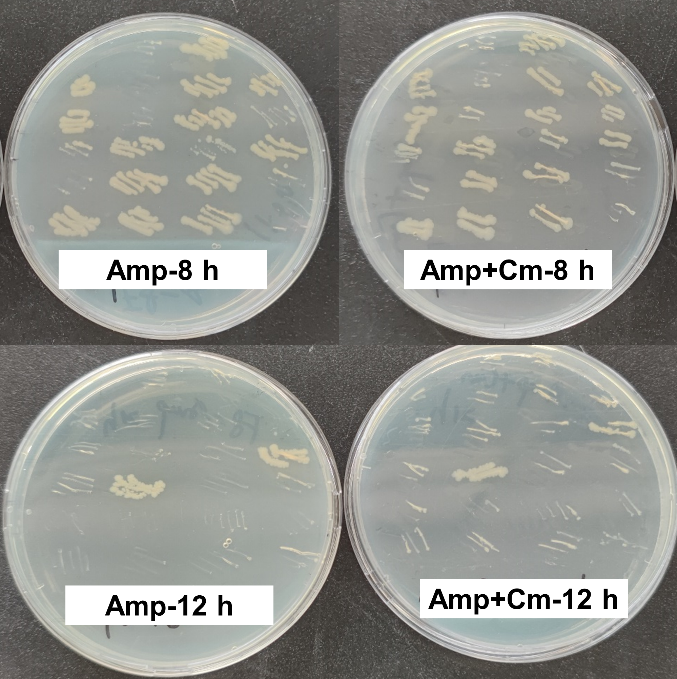
**

**Fig. S1** Verification of plasmid stability in strain ALA14 during fed-batch fermentation in 5 L bioreactors. Samples were taken at 8 h and 12 h to verify the plasmid stability. Amp means the LB solid plate with 100 μg/mL ampicillin. Amp + Cm means the LB solid plate with 100 μg/mL ampicillin and 20 µg/mL chloramphenicol.


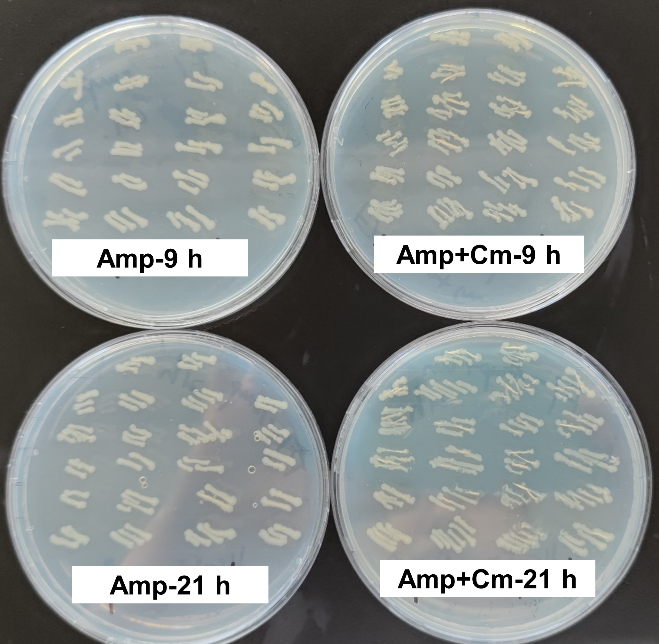


**Fig. S2** Verification of plasmid stability in strain ALA26 during fed-batch fermentation in 5 L bioreactors. Samples were taken at 9 h and 21 h to verify the plasmid stability. Amp means the LB solid plate with 100 μg/mL ampicillin. Amp + Cm means the LB solid plate with 100 μg/mL ampicillin and 20 µg/mL chloramphenicol.

**Table S1** Microbial production of 5-ALA by engineered *E. coli* and *C. glutamicum* strains via C4 biosynthetic pathway from different substrates

| **Strategy** | **Main substrates** | **Titer**  **(g/L)** | **Productivity (g/L∙h)** | **Reference** |
| --- | --- | --- | --- | --- |
| ***E.coli* strains** |  |  |  |  |
| Overexpression of *hemA* from *R. sphaeroides* | Succinate, glycine | 5.2 | 0.43 | [1] |
| Overexpression of *hemA* from *R. palustris* | Glucose, succinate, glycine | 5.2 | 0.32 | [2] |
| Overexpression of *hemA* from *R. sphaeroides* | Glucose, succinate, glycine | 6.6 | 0.24 | [3] |
| Overexpression of *hemA* from *Agrobacterium radiobacter* | Glucose, succinate, glycine, xylose | 7.3 | 0.24 | [4] |
| Overexpression of *hemO* from *R. palustris* | Glucose, succinate, glycine | 6.3 | 0.26 | [5] |
| Overexpression of *hemA* from *R. palustris*,  deletion of *sdhA* | Glucose, glycine | 6.4 | 0.30 | [6] |
| Overexpression of *hemA* from *A. radiobacter* | Glucose, succinate, glycine | 9.4 | 0.43 | [7] |
| Overexpression of *hemA* from *R. capsulatus* | Glycerol, glycine, succinate | 8.8 | 0.24 | [8] |
| Overexpression of *hemA* from *R. capsulatus*, native *coaA*^M^ and *serA*Δ*197*, repression of *hemB* by substituting the start codon AUG to GUG | Glucose | 2.8 | 0.06 | [9] |
| Overexpression of *hemA1* and *pgr7* from *Arabidopsis thaliana* | Glucose, glutamate | 7.6 | 0.16 | [10] |
| Overexpression of *hemA* from *R. palustris*, native *katE* and *sodB* | Glucose, glycine, succinate | 11.5 | 0.52 | [11] |
| Overexpression of *hemA* from *R. capsulatus* and GroELS chaperone | Glucose, glycine, succinate, PLP | 5.7 | 0.24 | [12] |
| Overexpression of *hemA* from *R. sphaeroides*, repression of *hemB* by CRISPRi, deletion of *ldhA*, *sdhA*, and *iclR* | Glycerol | 6.9 | 0.32 | [13] |
| Overexpression of *hemA* from *R. sphaeroides*, DnaK and GroELS chaperone, *rhtA*, *pdxK*, and *pdxY* | Glucose, glycine, succinate | 7.5 | 0.59 | [14] |
| Overexpression of *hemA* from *R. capsulatus*, GroELS chaperone and *pdxY* | Glucose, glycerol, succinate, glycine | 14.3 | 0.48 | [15] |
| Overexpression of *hemA* from *R. capsulatus* and GroELS chaperone | Glucose, glycerol, succinate, glycine | 15.6 | 0.56 | [16] |
| **Overexpression of *hemA*^C75A/R365K^ from *R. palustris*, native *coaA*, *ppc*, *eamA* and *grxA*, repression of *hemB*, *aceA* and *sucC* by synthetic sRNA** | **Glucose, glycine** | **30.7** | **1.02** | **This study** |
| ***C. glutamicum* strains** |  |  |  |  |
| Overexpression of *hemA* from *R. sphaeroides*, native *ppc*, and *rhtA* from *E. coli*, deletion of *ldhA*, *pqo*, *cat*, *pta*, *ackA*, and *pbp1b* | Glucose, glycine | 7.53 | 0.21 | [17] |
| Overexpression of ALAS from *R. capsulatus*, deletion of *sucCD* | Glucose, glycine | 7.60 | 0.09 | [18] |
| Overexpression of ALAS from *R. capsulatus* and *rhtA* from *E. coli*, deletion of *sucCD,* two-stage fermentation | Glucose, glycine | 14.7 | 0.92 | [18] |
| Overexpression of ALAS from *R. sphaeroides*, *serA*Δ*197*, *serB, serC* and *glyA* | Glucose, glycine | 3.40 |  | [19] |
| Overexpression of *hemA* from *R. palustris* and native *ppc* | Glucose, glycine | 16.3 | 0.42 | [20] |
| Overexpression of *hemA* from *R. palustris* and native *ppc* | Cassava bagasse  hydrolysate, glycine | 18.50 | 0.47 | [20] |
| Overexpression of *hemA* from *R. palustris*, and *eamA* from *E. coli*, deletion of *odhI* and *sdhA,* repression of *hemB* by synthetic sRNA | Glucose, glycine | 25.05 | 0.52 | [21] |

**Table S2** Bacterial strains and plasmids used in this study.

| **Strain or plasmid** | **Description** | **Reference or source** |
| --- | --- | --- |
| **Strains** |  |  |
| *E. coli* DH5α | F^−^ *supE*44 ∆*lacU*169 (φ80 *lacZ*∆M15) *hsdR*17 *recA*1 *endA*1 *gyrA*96 thi-1 *relA*1 | Invitrogen |
| *E. coli* MG1655 | Wild type | Lab stock |
| ZLEcA4 | MG1655 (∆*coaA*) containing plasmid pZPA43 | This study |
| ALA1 | *E. coli* MG1655 harboring plasmid pZGA24 and pZCA9 | This study |
| ALA2 | *E. coli* MG1655 harboring plasmid pZGA24 and pZSA13 | This study |
| ALA3 | *E. coli* MG1655 harboring plasmid pZGA24 and pZSA14 | This study |
| ALA4 | *E. coli* MG1655 harboring plasmid pZGA24 and pZSA41 | This study |
| ALA5 | *E. coli* MG1655 harboring plasmid pZGA24 and pZCA134 | This study |
| ALA6 | *E. coli* MG1655 harboring plasmid pZGA24 and pZCA135 | This study |
| ALA7 | *E. coli* MG1655 harboring plasmid pZGA24 and pZCA137 | This study |
| ALA8 | *E. coli* MG1655 harboring plasmid pZGA24 and pZCA136 | This study |
| ALA9 | *E. coli* MG1655 harboring plasmid pZPW70 and pZCA136 | This study |
| ALA10 | *E. coli* MG1655 harboring plasmid pZPW71 and pZCA136 | This study |
| ALA11 | *E. coli* MG1655 harboring plasmid pZPW72 and pZCA136 | This study |
| ALA12 | *E. coli* MG1655 harboring plasmid pZPW73 and pZCA136 | This study |
| ALA13 | *E. coli* MG1655 harboring plasmid pZPW74 and pZCA136 | This study |
| ALA14 | *E. coli* MG1655 harboring plasmid pZPW76 and pZCA136 | This study |
| ALA15 | *E. coli* MG1655 harboring plasmid pZPW77 and pZCA136 | This study |
| ALA16 | *E. coli* MG1655 harboring plasmid pZPW78 and pZCA136 | This study |
| ALA17 | *E. coli* MG1655 harboring plasmid pZPW79 and pZCA136 | This study |
| ALA18 | *E. coli* MG1655 harboring plasmid pZPW80 and pZCA136 | This study |
| ALA19 | *E. coli* MG1655 harboring plasmid pZPW81 and pZCA136 | This study |
| ALA20 | *E. coli* MG1655 harboring plasmid pZPW82 and pZCA136 | This study |
| ALA21 | *E. coli* MG1655 harboring plasmid pZPW83 and pZCA136 | This study |
| ALA22 | *E. coli* MG1655 harboring plasmid pZPW84 and pZCA136 | This study |
| ALA23 | *E. coli* MG1655 harboring plasmid pZPW85 and pZCA136 | This study |
| ALA24 | *E. coli* MG1655 harboring plasmid pZPW86 and pZCA136 | This study |
| ALA25 | *E. coli* MG1655 harboring plasmid pZPW87 and pZCA136 | This study |
| ALA26 | *E. coli* MG1655 (∆*coaA*) harboring plasmid pZPW76 and pZCA136 | This study |
| **Plasmids** |  |  |
| pTrc99A | Expression vector of *E. coli*, IPTG-inducible promoter *P_trc_*, Ampicillin-resistant (Amp^R^) | [22] |
| pWSK29 | Cloning vector of *E. coli*, pSC101 origin, Amp^R^ | [23] |
| pEASY-Blunt | Cloning vector, pUC Origin, Kan^R^, Amp^R^ | TransGen Biotech |
| pSB4C5 | Cloning vector, Cm^R^ | [24] |
| pACYC184 | Cloning vector, Tet^R^ | [25] |
| pUC57-sRNA | Cloning vector, pUC Origin, kan^R^, sR*sucC* | This study |
| pZPA17 | pEASY-Blunt containing the *coaA* gene and its upstream 500 bp and downstream 500 bp | This study |
| pZPA18 | pEASY-Blunt containing upstream 500 bp and downstream 500 bp of *coaA* gene | This study |
| pZPA19 | pEASY-Blunt containing upstream 500 bp of *coaA* gene, *cm-sacB* and downstream 500 bp of *coaA* gene | This study |
| pKD46 | Amp^R^, helper plasmid | [26] |
| pDS132 | *Cm*-resistant gene and selection marker *sacB* gene | [27] |
| pZPA43 | pKD46 carrying *coaA* promoer and *coaA* gene | This study |
| pZCA9 | Cm^R^, pSC101 replicon | This study |
| pZCA9p | Deletion of the *lac* promoter in pZCA9 | This study |
| pTrc99A-*hemA* | pTrc99A carring *hemA* gene from *Rhodopseudomonas*  *palustris* ATCC 17001 | [5] |
| pET21a–*hemA* | pET21a containing *hemA* from *R. palustris* ATCC 17001 | [5] |
| pZSA13 | pZCA9p containing sR*hemB* gene, the promoter of *P_tet_* and the terminator of T1/TE | This study |
| pZSA14 | pZCA9p containing sR*sucC* gene, the promoter of *P_tet_* and the terminator of T1/TE | This study |
| pZSA41 | pZCA9p containing sR*aceA* gene, the promoter of *P_tetO_* and the terminator of T1/TE | This study |
| pZCA134 | pZCA9p containing sR*sucC* and sR*hemB* gene, the promoter of *P_tet_* and the terminator of T1/TE | This study |
| pZCA135 | pZCA9p containing sR*sucC* and sR*aceA* gene, the promoter of *P_tet_* and the terminator of T1/TE | This study |
| pZCA136 | pZCA9p containing sR*sucC*, sR*hemB* and sR*aceA* gene, the promoter of *P_tet_* and the terminator of T1/TE | This study |
| pZCA137 | pZCA9p containing sR*hemB* and sR*aceA* gene, the promoter of *P_tet_* and the terminator of T1/TE | This study |
| pZPW70 | pZGA24 containing *P*_J23100_ promoter and *coaA* gene | This study |
| pZPW71 | pZPW70 containing *P_ppc_* promoter and *ppc* gene | This study |
| pZPW72 | pTrc99A carring *hemA*^C75A/R365K^ gene, *P*_J23100_ and *coaA* gene, and *P_ppc_* promoter and *ppc* gene | This study |
| pZPW73 | pZPW72 containing *P_rhtA_* and *rhtA* gene | This study |
| pZPW74 | pZPW72 containing *P_eamA_* and *eamA* gene | This study |
| pZPW76 | pZPW74 containing *P_grxA_* and *grxA* gene | This study |
| pZPW77 | pZPW74 containing *P_trxC_* and *trxC* gene | This study |
| pZPW78 | pZPW74 containing *P_msrA_* and *msrA* gene | This study |
| pZPW79 | pZPW74 containing *P_msrB_* and *msrB* gene | This study |
| pZPW80 | pZPW74 containing *P_katE_* and *katE* gene | This study |
| pZPW81 | pZPW74 containing *P_katG_* and *katG* gene | This study |
| pZPW82 | pZPW74 containing *P_sodA_* and *sodA* gene | This study |
| pZPW83 | pZPW74 containing *P_sodB_* and *sodB* gene | This study |
| pZPW84 | pZPW74 containing *P_sodC_* and *sodC* gene | This study |
| pZPW85 | pZPW76 containing *P_katG_* and *katG* gene | This study |
| pZPW86 | pZPW76 containing *P_sodA_* and *sodA* gene | This study |
| pZPW87 | pZPW76 containing *P_sodC_* and *sodC* gene | This study |

**Table S3** Primers used in this study.

| **Primers** | **Sequence(5’-3’)** | **Relevance** |
| --- | --- | --- |
| pKD46-*coaA*-F | CAAATAGGGGTTCCGCGCACTCAACGATAGCTTCCTGGCAGAGAT | pZPA43 construction |
| pKD46-*coaA*-R | GTGGCACTTTTCGGGGAAATTTATTTGCGTAGTCTGACCT |  |
| pKD46-Rev-F | ATTTCCCCGAAAAGTGCCACCTG |  |
| pKD46-Rev-R | GTGCGCGGAACCCCTATTTGTT |  |
| *coaA*-500-F | AGTCTGTGACGGCAGATTTA | pZPA18 and pZPA19 construction |
| *coaA*-500-R | TGCAGATAAAGTTCGTGTTAA |  |
| *coaA*-Rev-F | TTTGCAGGGGAGCGAATACT |  |
| *coaA*-Rev-R | GGTTTAGCAGGAGCGCATTG |  |
| *cm*-F | GGTGAAAACCTCTGACACATGCAG |  |
| *sacB*-R | CGCCAGTGTGCTGGAATTGC |  |
| *ppc*-F | CCGCAAGCTTTATCCGACCTACACCTTTGGT |  |
| *ppc*-R | CCGCAAGCTTGGACTTCTGTGGAATGCATAGT |  |
| pZCA9-F | GCCTGGGGTGCCTAATGAGT | pZCA9p construction |
| pZCA9-R | TCACACAGGAAACAGCTATGAC |  |
| TsR-F | TCCCTATCAGTGATAGAGATTGAC | pZSA14 construction |
| TsR-R | GAGAGCGTTCACCGACAAACA |  |
| *tetR*-F | TTGTCGGTGAACGCTCTCGGGTCGACTCTAGAGGATCT |  |
| *tetR*-R | CCTGATTCTGTGGATAACCGGCTTTTAAGACCCACTTTCA |  |
| p-F | CGGTTATCCACAGAATCAGG |  |
| p-R | TCTCTATCACTGATAGGGACGCCTTTGAGTGAGCTGATAC |  |
| sR*hemB*-F | TAAGTCTGTCATTTTCTGTTGGGCCATTGCAT | pZSA13 construction |
| sR*hemB*-R | ATCCAACGCCCTGTGCTCAGTATCTCTATCACTGA |  |
| sR*aceA*-F | ACGGGTTTTCATTTTCTGTTGGGCCATTGCAT | pZSA41 construction |
| sR*aceA*-R | ACACAACAAATTGTGCTCAGTATCTCTATCACTGA |  |
| TsRBC-F | TTTGTCGGTGAACGCTCTCTAATGAATCGGCCAACGCG | pZCA134 construction |
| TsRBC-R | TCTCTATCACTGATAGGGATAATGCAGCTGGCACGACA |  |
| TsRAC-F | TTTGTCGGTGAACGCTCTCAGCGGTATCAGCTCACTCAA | pZCA135 and pZCA136 construction |
| TsRAC-R | TCTCTATCACTGATAGGGACCAATACGCAAACCGCCTCT |  |
| TsRAB-F | GGGTCGACTCTAGAGGATCT | pZCA137 construction |
| TsRAB-R | CGCCTTTGAGTGAGCTGATAC |  |
| pTrc99a-Rev-2F | GTCAGAGGTTTTCACCGTCAT | pZPW70 construction |
| pTrc99a-Rev-2R | ACAGCTTGTCTGTAAGCGGA |  |
| *coaA-*F | TCCGCTTACAGACAAGCTGTTTATTTGCGTAGTCTGACCTCTTC |  |
| *coaA-*R | TGACGGTGAAAACCTCTGACTTGACGGCTAGCTCAGTCCT |  |
| *hemA*-F | ACCAATGCTTCTGGCGTCA |  |
| *ppc*-R | CAGACCGCTTCTGCGTTCT |  |
| *amp*-F | AACAGAATTTGCCTGGCGG |  |
| *lacI*-R | TGACGCCAGAAGCATTGGT |  |
| *rhtA*-F | CAGAACGCAGAAGCGGTCTGTCAGGTTGAAACCGCCCA | pZPW73 construction |
| *rhtA-*R | GCCGCCAGGCAAATTCTGTTCGCCAGGAATTTTCGGAA |  |
| *eamA-*F | CAGAACGCAGAAGCGGTCTGAAAGAGCACAACCAGCGTCA | pZPW74 construction |
| *eamA-*R | GCCGCCAGGCAAATTCTGTTTTAACTTCCCACCTTTACCGCT |  |
| *eamA*-2R | TTAACTTCCCACCTTTACCGCT |  |
| *grxA*-F | CGGTAAAGGTGGGAAGTTAATCATTCTGGCAAGAGCTGGC | pZPW76 construction |
| *grxA*-R | GCCGCCAGGCAAATTCTGTTACCTGAAGAATAACCACCACCG |  |
| *trxC*-F | CGGTAAAGGTGGGAAGTTAATTCGGTCATAAGAGAGCGTCG | pZPW77 construction |
| *trxC*-R | GCCGCCAGGCAAATTCTGTTGGCTGACTCTGTTTGTCATCG |  |
| *msrA*-F | CGGTAAAGGTGGGAAGTTAAATGCAAAACTGCCTGATACGC | pZPW78 construction |
| *msrA*-R | GCCGCCAGGCAAATTCTGTTGATAACCCCTCGACCTGTAGAC |  |
| *msrB*-F | CGGTAAAGGTGGGAAGTTAAGCATCATGCTGTTGATAATGTC | pZPW79 construction |
| *msrB*-R | GCCGCCAGGCAAATTCTGTTTGAGCAGCACGGAAAACA |  |
| *katE-*F | CGGTAAAGGTGGGAAGTTAACGGCATAAACAAAGCGCAC | pZPW80 construction |
| *katE*-R | GCCGCCAGGCAAATTCTGTTAGCGGTTTCTTCATTACTGGC |  |
| *katG*-F | CGGTAAAGGTGGGAAGTTAAGAACGTTCAGCGACTGCC | pZPW81 construction |
| *katG*-R | GCCGCCAGGCAAATTCTGTTTGAGTTACGCGATTTGCCAT |  |
| *sodA*-F | CGGTAAAGGTGGGAAGTTAAAGCTGATATGCGGCCTATACG | pZPW82 construction |
| *sodA*-R | GCCGCCAGGCAAATTCTGTTTGCCAAAATATCCCCATCG |  |
| *sodB*-F | CGGTAAAGGTGGGAAGTTAACGATTTATCTTGCCGGATGC | pZPW83 construction |
| *sodB*-R | GCCGCCAGGCAAATTCTGTTGCGCACAGGTCAGGAAATT |  |
| *sodC*-F | CGGTAAAGGTGGGAAGTTAACAATTGTCTGGCGGCTGTAC | pZPW84 construction |
| *sodC*-R | GCCGCCAGGCAAATTCTGTTGTATTACGTTTACCATCGCAGCC |  |
| *grxA-*2F | ACCTGAAGAATAACCACCACCG | pZPW85, pZPW86 and pZPW87 construction |
| *grxA-*2R | GCCGCCAGGCAAATTCTGTTTCATTCTGGCAAGAGCTGGC |  |
| *katG-*2R | GTGGTGGTTATTCTTCAGGTTGAGTTACGCGATTTGCCAT |  |
| *sodA-*2R | GTGGTGGTTATTCTTCAGGTTGCCAAAATATCCCCATCG |  |
| *sodC-*2R | GTGGTGGTTATTCTTCAGGTGTATTACGTTTACCATCGCAGCC |  |
| *coaA*-DF | AGTCTGTGACGGCAGATTTA | Construction of strain ZLEcA4 |
| *coaA*-DR | TGCAGATAAAGTTCGTGTTAA |  |
| *coaA*-TF | GAGAAGCTCTCTTTATTCGAGCCG |  |
| *coaA*-TR | ATTTGTCGATGTTCTGGCGTCTGG |  |
| C51A-F | CGGTGTGGGCCTCCAACGATTATCTCGGC | pET21a(+)-*hemA*^C51A^ construction |
| C51A-R | CGTTGGAGGCCCACACCGTCACATCGCCG |  |
| C75A-F | GACAGCGCCGGCGCCGGCGCCGGCGGCAC | pET21a(+)-*hemA*^C75A^ construction |
| C75A-R | GCCGGCGCCGGCGCTGTCCAGCGCCTCGTG |  |
| C132A-F | CATGCCCGGCGCCGTGATCCTGTCGGACG | pET21a(+)-*hemA*^C132A^ construction |
| C132A-R | GATCACGGCGCCGGGCATGCGCGACGCCAGC |  |
| C200A-F | CGAAATCGCCGACGTCGCCGACGCGCATAACG | pET21a(+)-*hemA*^C200A^ construction |
| C200A-R | CGTCGGCGACGTCGGCGATTTCGGCGATCGGC |  |
| C263A-F | CTCGGCCGTCGCCGATTTCGTCCGCAGCTTCG | pET21a(+)-*hemA*^C263A^ construction |
| C263A-R | GAAATCGGCGACGGCCGAGGAGCCGGCGATG |  |
| C340A-F | GCAGCGCTGGCCAAGCAGATCAGCGACGAG | pET21a(+)-*hemA*^C340A^ construction |
| C340A-R | GATCTGCTTGGCCAGCGCTGCATCGCCGAC |  |
| P357T-F | GTGCAGACCATCAACTATCCGACCGTG | pET21a(+)-*hemA*^P357T^ construction |
| P357T-R | GATAGTTGATGGTCTGCACATAGATGCCGTAC |  |
| R365K-F | GTGCCGAAAGGCACCGAGCGCCTTCGGATCAC | pET21a(+)-*hemA*^R365K^ construction |
| R365K-R | CGGTGCCTTTCGGCACGGTCGGATAGTTGATC |  |

**Table S4** Binding sequences of sRNAs used in this study.

| **Target gene** | **24-mer target binding sequence of sRNA** |
| --- | --- |
| *sucC* | TGCCTGATATTCATGTAAGTTCAT |
| *aceA* | ACGTGGCTTTCCAACGTTTGACAT |
| *hemB* | AGGGCGTTGGATTAAGTCTGTCAT |

**References**

1. Xie L, Hall D, Eiteman MA, Altman E. Optimization of recombinant aminolevulinate synthase production in *Escherichia coli* using factorial design. Appl Microbiol Biotechnol. 2003;63:267-273.

2. Choi HP, Lee YM, Yun CW, Sung HC. Extracellular 5-aminolevulinic acid production by *Escherichia coli* containing the *Rhodopseudomonas palustris* KUGB306 *hemA* gene. J Microbiol Biotechnol. 2008;18:1136-1140.

3. Fu W, Lin J, Cen P. Enhancement of 5-aminolevulinate production with recombinant *Escherichia coli* using batch and fed-batch culture system. Bioresour Technol. 2008;99:4864-4870.

4. Lin J, Fu W, Cen P. Characterization of 5-aminolevulinate synthase from *Agrobacterium radiobacter*, screening new inhibitors for 5-aminolevulinate dehydratase from *Escherichia coli* and their potential use for high 5-aminolevulinate production. Bioresour Technol. 2009;100:2293-2297.

5. Zhang L, Chen J, Chen N, Sun J, Zheng P, Ma Y. Cloning of two 5-aminolevulinic acid synthase isozymes HemA and HemO from *Rhodopseudomonas palustris* with favorable characteristics for 5-aminolevulinic acid production. Biotechnol Lett. 2013;35:763-768.

6. Pu W, Chen J, Sun C, Chen N, Sun J, Zheng P, Ma Y. Deficiency of succinic dehydrogenase or succinyl-coA synthetase enhances the production of 5-aminolevulinic acid in recombinant *Escherichia coli*. Sheng Wu Gong Cheng Xue Bao. 2013;29:1494-1503.

7. Yang J, Zhu L, Fu WQ, Lin YJ, Lin JP, Cen PL. Improved 5-aminolevulinic acid production with recombinant *Escherichia coli* by a short-term dissolved oxygen shock in fed-batch fermentation. Chinese J Chem Eng. 2013;21:1291–1295.

8. Lou JW, Zhu L, Wu MB, Yang LR, Lin JP, Cen PL. High-level soluble expression of the *hemA* gene from *Rhodobacter capsulatus* and comparative study of its enzymatic properties. J Zhejiang Univ Sci B. 2014;15:491-499.

9. Ding W, Weng H, Du G, Chen J, Kang Z. 5-Aminolevulinic acid production from inexpensive glucose by engineering the C4 pathway in *Escherichia coli*. J Ind Microbiol Biotechnol. 2017;44:1127-1135.

10. Aiguo Z, Meizhi Z. Production of 5-aminolevulinic acid from glutamate by overexpressing *HemA1* and *pgr7* from *Arabidopsis thaliana* in *Escherichia coli*. World J Microbiol Biotechnol. 2019;35:175.

11. Zhu C, Chen J, Wang Y, Wang L, Guo X, Chen N, Zheng P, Sun J, Ma Y. Enhancing 5-aminolevulinic acid tolerance and production by engineering the antioxidant defense system of *Escherichia coli*. Biotechnol Bioeng. 2019;116:2018-2028.

12. Yu TH, Yi YC, Shih IT, Ng IS. Enhanced 5-aminolevulinic acid production by co-expression of codon-optimized *hemA* gene with chaperone in genetic engineered *Escherichia coli*. Appl Biochem Biotechnol. 2020;191:299-312.

13. Miscevic D, Mao JY, Kefale T, Abedi D, Moo-Young M, Perry Chou C. Strain engineering for high-level 5-aminolevulinic acid production in *Escherichia coli*. Biotechnol Bioeng. 2021;118:30-42.

14. Shih IT, Yi YC, Ng IS. Plasmid-free system and modular design for efficient 5-aminolevulinic acid production by engineered *Escherichia coli*. Appl Biochem Biotechnol. 2021;193:2858-2871.

15. Yi Y-C, Xue C, Ng IS. Low-carbon-footprint production of high-end 5-aminolevulinic acid via integrative strain engineering and RuBisCo-equipped *Escherichia coli*. ACS Sustainable Chem Eng. 2021;9:15623-15633.

16. Yu T-H, Tan S-I, Yi Y-C, Xue C, Ting W-W, Chang J-J, Ng IS. New insight into the codon usage and medium optimization toward stable and high-level 5-aminolevulinic acid production in *Escherichia coli*. Biochem Eng J. 2022;177:108259.

17. Feng L, Zhang Y, Fu J, Mao Y, Chen T, Zhao X, Wang Z. Metabolic engineering of *Corynebacterium glutamicum* for efficient production of 5-aminolevulinic acid. Biotechnol Bioeng. 2016;113:1284-1293.

18. Yang P, Liu W, Cheng X, Wang J, Wang Q, Qi Q. A new strategy for production of 5-aminolevulinic acid in recombinant *Corynebacterium glutamicum* with high yield. Appl Environ Microbiol. 2016;82:2709-2717.

19. Zou Y, Chen T, Feng L, Zhang S, Xing D, Wang Z. Enhancement of 5-aminolevulinic acid production by metabolic engineering of the glycine biosynthesis pathway in *Corynebacterium glutamicum*. Biotechnol Lett. 2017;39:1369-1374.

20. Chen J, Wang Y, Guo X, Rao D, Zhou W, Zheng P, Sun J, Ma Y. Efficient bioproduction of 5-aminolevulinic acid, a promising biostimulant and nutrient, from renewable bioresources by engineered *Corynebacterium glutamicum*. Biotechnol Biofuels. 2020;13:41.

21. Wang L, Yan S, Yang T, Xu M, Zhang X, Shao M, Li H, Rao Z. Engineering the C4 pathway of *Corynebacterium glutamicum* for efficient production of 5-aminolevulinic acid. Sheng Wu Gong Cheng Xue Bao. 2021;37:4314-4328.

22. Amann E, Ochs B, Abel KJ. Tightly regulated tac promoter vectors useful for the expression of unfused and fused proteins in *Escherichia coli*. Gene. 1988;69:301-315.

23. Wang RF, Kushner SR. Construction of versatile low-copy-number vectors for cloning, sequencing and gene expression in *Escherichia coli*. Gene. 1991;100:195-199.

24. Pandi A, Koch M, Voyvodic PL, Soudier P, Bonnet J, Kushwaha M, Faulon JL. Metabolic perceptrons for neural computing in biological systems. Nat Commun. 2019;10:3880.

25. Bahk JD, Sakai H, Komano T. Plasmid pACYC184 contains an *ssi* signal for initiation of single-strand phage DNA replication. Gene. 1988;65:93-99.

26. Datsenko KA, Wanner BL. One-step inactivation of chromosomal genes in *Escherichia coli* K-12 using PCR products. Proc Natl Acad Sci U S A. 2000;97:6640-6645.

27. Philippe N, Alcaraz JP, Coursange E, Geiselmann J, Schneider D. Improvement of pCVD442, a suicide plasmid for gene allele exchange in bacteria. Plasmid. 2004;51:246-255.
